# DyeSPY: Establishing the First Forensic SERS Reference for Hair Dye Colorant Evidence

**DOI:** 10.1021/acs.analchem.5c05023

**Published:** 2025-11-28

**Authors:** Aidan P. Holman, Avery Maalouf, Dmitry Kurouski

**Affiliations:** † Department of Biochemistry and Biophysics, 14736 Texas A&M University, College Station, Texas 77843, United States; ‡ Interdisciplinary Faculty of Toxicology, 14736 Texas A&M University, College Station, Texas 77843, United States

## Abstract

Hair dyeing is a widespread practice with potential forensic value in individual identification, yet most analytical approaches are destructive, time-intensive, or lack sensitivity for trace residues. Surface-enhanced Raman spectroscopy (SERS) offers a rapid, nondestructive, and highly sensitive alternative. We introduce DyeSPY, the first forensic SERS and machine learning platform for identifying oxidative and nonoxidative hair dye colorants and predicting their perceptual colors. Using spectra from 44 pure colorants, laboratory-prepared mixtures, and 60 commercial dye products applied to hair, we developed a three-phase classification pipeline. Phase I distinguished oxidative from nonoxidative dyes with up to 98.6% accuracy on hair using partial least-squares discriminant analysis. Phase II achieved high-fidelity colorant identification: for nonoxidative dyes, synthetic training via linear spectral mixing yielded an *F*1 score of 0.88 with 85.7% mean subset recall; for oxidative dyes, an artificial neural network attained perfect hair classification (*F*1 = 1.00) and 98.5% subset recall for dye solutions. Phase III predicted perceptual colors with ≥97.5% accuracy by using cosine similarity. Validation on external data sets confirmed robust performance despite substrate variability. By integrating chemically informed modeling of stable and reactive dye systems, DyeSPY establishes a forensic-grade framework for accurate and interpretable hair dye analysis.

## Graphical abstract

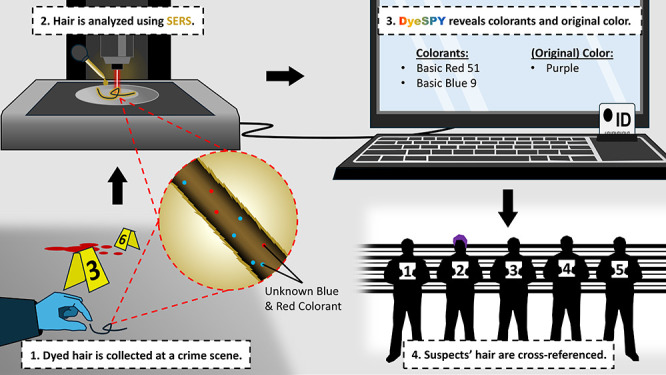

## 1 Introduction

Colorants are chemicals that produce or contribute to color in dyes, which may contain one or more such compounds. They fall into four main categories: primary intermediates, couplers, pigments, and direct dyes. Primary intermediates, containing aromatic rings and amine groups, require oxidation to quinoid or imine forms to generate color. Couplers react with intermediates to produce new hues or modify tones, while pigments alter color without reacting. Direct dyes impart color independently, without oxidation or chemical bonding. Permanent and demi-permanent dyes typically combine primaries and couplers (and sometimes pigments), whereas semipermanent and temporary dyes rely largely on direct dyes. The main distinction between pigments and direct dyes lies in binding: pigments do not necessarily bond strongly to substrates.

The practice of hair dyeing is common among both men and women, with prevalence rates ranging from 11 to 48% among adult men in North America and 50 to 80% among adult women in the United States (US), Japan, and the European Union (EU).^1,2^ While often used as a form of self-expression, many individuals continue dyeing despite adverse reactions, suggesting that, for many, hair coloring is viewed as essential.^3^ Given its widespread use and significance for personal identity, hair dye may have potential applications in forensic science, particularly in aiding suspect-to-perpetrator distillation.

Forensic hair analysis generally relies on light microscopy to determine the racial origin, treatment, and other characteristics of hair, including the color of dye, if applicable.^4^ In fact, dyed/bleached hair is said to be more useful to the examiner than untreated hair evidence.^5^ However, dye classification in this manner faces high subjectivity and, as purported by a popular handbook, requires the presence of a follicular root, which is rapidly degraded (if present) after leaving the scalp.^4,5^ This weakness has been treated, with varying levels of “success”, by several spectral techniques such as high-performance thin-layer chromatography (HPTLC), X-ray fluorescence (XRF), and ultraviolet–visible (UV–vis) spectroscopy.^6–8^ These techniques can be used to reveal the color or colorants from the hair cuticle using methods such as characteristic peaks and artificial intelligence, reducing cognitive bias. However, the most widely recognized techniques for individualistic characterization of dyes are infrared (IR) and Raman (micro)­spectroscopy (RS), given the existence of validated, large reference libraries.^9^ Many of these techniques fall short of detecting very minute quantities of colorants or pigments.^6,9,10^

Fortunately, surface-enhanced RS (SERS) has reported success at individualizing colorants in extremely low concentrations, even for single molecules.^11^ This is accomplished by the use of plasmonic nanoparticles, made of metals, that enhance the signal of conventional Raman spectra by about a million-fold.^12^ SERS, in turn, shows promise for detecting extremely degraded colorants, which forensic analysts may require.^13–18^ However, current SERS-based analysis of hair dyes focuses on the classification of the hair dye product itself, i.e., the overall dye signal and not the individual colorant(s). This fails to consider that companies may change their ingredients whenever they wish. Not to mention that prolonged degradation of colorants in dyes on hair lowers the probability of their differentiation among other dyes.^14^ Therefore, the prediction and consequent comparison of colorants within dyes on hair become more realistic.

One of the most sophisticated databases for colorant-specific detection, developed by Palenik and co-workers in 2011, are search-based libraries that allow the user to match reference spectra with unknown samples.^9^ A fundamental limitation of this database, as indicated by both its structural composition and discussion, is that it is primarily tailored toward fabric analysis. This focus is underscored by a critical omission: the absence of couplers. Couplers, essential chemical agents in oxidative dye formulations, bind to primary intermediates under oxidative conditions, initiating polymerization and thereby altering the molecular structure to generate new chromophores. The significance of couplers cannot be overstated, given that they are fundamental constituents of permanent hair dye formulations—products that dominate nearly 80% of the global hair dye market.^19^ Consequently, any database designed to accurately identify hair dyes must integrate the inherent chemical transformations induced by couplers, ensuring a more comprehensive and chemically rigorous approach to forensic dye analysis.

Furthermore, identifying hazardous colorants in hair dyes is critical for assessing toxicological risk. Certain colorants found in hair dyes can be classified as mutagenic, genotoxic, carcinogenic, and skin and eye irritants.^20^ The U.S. Food and Drug Administration (FDA), under the Federal Food, Drug, and Cosmetic Act of 1938 (FD&C Act), restricts all colorants added to cosmetics, including hair dyes.^21^ However, to date, the FDA has only banned one colorant (previously) used in hair dye products: lead acetate.^22^ In contrast, the European Union had, as of 2017, banned nearly 50 colorants and imposed restrictions on more than 50 others.^23,24^ This regulatory gap highlights a concerning issue—colorants banned for safety reasons in the EU may still be legally used in products sold in the U.S., posing ongoing health risks to American consumers. For instance, many salons’ stylists mix hair dyes together to achieve their clients’ desired hair color.^25^ Thus, identifying a single hair dye becomes problematic in this scenario, and identifying the specific colorants that may have elicited the adverse reaction becomes more useful.

The goal of this study is to use SERS and machine learning to build a highly accurate database for the identification of colorants in hair dyes. The database will be designed to output possible colorants within a tested sample, as well as the possible colors declared by commercially available dye products. The described database has the potential to aid forensic investigations for victim and perpetrator/suspect identification, as well as for identifying hazardous colorant ingredients in hair dyes for toxicological findings.

## 2 Materials and Methods

### 2.1 Colorants

A total of 44 colorants were purchased from several suppliers and were used as received (see Section 2.2 for details on how each colorant was used as samples). Table S1 reflects all colorants used throughout this experiment, including their names, Chemical Abstracts Service (CAS) number, supplier, whether they are considered oxidative or nonoxidative, type of colorant, and our experiment-specific identification (ESID) code.

Some colorants, such as 3-nitro-*p*-hydroxyethylaminophenol and 2-amino-6-chloro-4-nitrophenol, can act as primaries, couplers, or direct dyes, depending on the conditions. In oxidative environments, they may react with other intermediates via hydroxyl or amine groups or oxidize independently to form quinonoid or imine-like structures that enhance conjugation and charge transfer. In nonoxidative settings, their amino and nitro substituents alone generate internal charge transfer, producing visible color through altered electronic transitions. Although such compounds exhibit multifunctional behavior, they are categorized here as oxidative couplers, since they are typically formulated alongside other oxidative dyes. The implications of this classification are discussed in Section 3.2.

### 2.2 Sample Preparation

#### 2.2.1 Model Training Samples

Oxidative hair dyes consist of approximately 80% of hair dye market shares in the EU and US.^19^ Accordingly, we calibrated the model using hair dyes (D1-D60) representative of commercially available formulations, with 73.3% corresponding to permanent and demi-permanent dyes (44 products) and the remaining 26.7% to semipermanent dyes (16 products) (Table S2). The hair dyes chosen this way were restricted to those for which we possessed colorants for.

All colorants were initially diluted to 20% (w/v) solutions in Type I ultrapure water, and a 20% (w/v) hydrogen peroxide solution was subsequently prepared. Samples of single colorants found in Table S1 were prepared by diluting each to 2% (w/v) in Type I ultrapure water. In other words, 2 μL of colorant (20%), 2 μL of H_2_O_2_ (20%), and 16 μL of water were used for primary intermediates, and 2 μL of colorant and 18 μL of water were used for direct dyes. For couplers, which do not give color on their own but rather alter the absorption of primary intermediates, these were prepared by themselves since Raman can detect organic dyes and also by mixing one with each primary intermediate for a total of 110 additional samples (5 primary intermediates × 22 couplers). Samples prepared this way consisted of 2 μL of primary intermediate solution, 2 μL of coupler solution, 2 μL of H_2_O_2_ solution, and 14 μL of water.

**Table 1:** Arsenal of Machine Learning Models, Their Type of Feature Discrimination, and a Short Summary of How They Work

model	model type	mode of function
LRDA	linear	learns a linear decision boundary by estimating class probabilities using logistic regression
PLSDA	linear	projects data onto latent variables that maximize covariance with class labels, then classifies based on linear separation
RFDA	nonlinear	constructs an ensemble of decision trees; classifies based on majority vote across trees trained on random feature subsets
XGBDA	nonlinear	builds an additive model of boosted decision trees to minimize classification loss and capture complex feature interactions
ANNDA	nonlinear	uses a multilayer neural network to learn hierarchical nonlinear representations for class separation
CSNNC	nonparametric	assigns class labels based on cosine angle similarity to training instances

#### 2.2.2 Model Validation Samples

Colorants found in D1–D60 were mixed appropriately to optimize our database (validation) in preparation for real product sample testing. All validation samples (artificially prepared dyes, a-[D#]) were made by bringing the final concentration of appropriate colorant(s) from D1–D60 to 2% each; additionally, hydrogen peroxide was added to reach 2% as well (if applicable). It should be noted that not all colorants in Table S1 will be utilized this way. This work is intended to further gauge the specificity of our database.

#### 2.2.3 Model Testing Samples

The models reported here were tested by analyzing hair dyes from Table S2 (i) in solution (sD1–sD60) and (ii) for their intended purposes: on hair (hD1–hD60). This was done for all oxidative hair dyes by mixing the dye and Ion Sensitive Scalp 20-grade developer at a 1:1 ratio and carefully massaging the mixture into virgin, unbleached, light-blonde hair from an unknown source. Hair collected this way came from a volunteer at a local salon shop. The volunteer collected hair from a customer from the apron, and it was rinsed with distilled water before use. SERS analysis was conducted on the undyed hair to visually confirm the absence of colorants through spectral output (Figure S1). All semipermanent dyes were used directly on the hair and gently massaged by hand. The dyes were allowed to rest on the hair according to package instructions (varied). After the allotted time, excess dye was rinsed off under cold, low-pressure tap water (to mimic real dyeing practices) by using a small-pore strainer and left to air-dry at room temperature before analysis.

**Table 2:** Hyperparameters Chosen and Respective Model Metrics for Phase I Validation Using aD1–aD60^a^

model	hyperparameters	global F1 score, %	accuracy, %	oxidative classification sensitivity, %	nonoxidative classification sensitivity, %	MCC
LRDA	C: 100	98.40	98.4	98.96	96.15	0.9566
	Max_iter: 500					
	penalty: L2					
PLSDA	LVs: 17	100	100	100	100	1.00
RFDA	Max_depth: 5	99.6	99.6	100	98.0	0.9886
	Min_samples_split: 10					
	N_estimators: 100					
XGBDA	eta: 0.05	99.2	99.2	99.49	98.0	0.9772
	Max_depth: 1					
	N_estimators: 500					
ANNDA	dropout: 0	78.0	78.0	100	0.00	0.00
	optimizer: SGD					
	eta: 0.01					
	batch size: 32					
	L2:0					
	L1:0					
CSNNC	N/A	100	100	100	100	1.00

a N/A: not applicable.

The solutions of all dyes, sD1–sD60, were sampled by adding a drop (or drop-size) of the dye to 200 μL of ultrapure water, as well as a drop of developer, if applicable. These samples were then vigorously mixed for 30s by trituration and left to react for 1 h.

### 2.3 Gold Nanorod Synthesis

Chemicals utilized for synthesis were: Milli-Q ultrapure water, type I (H_2_O), cetyltrimethylammonium bromide (CTAB, VWR International), gold­(III) chloride hydrate (HAuCl_4_·H_2_O, Aldrich), ascorbic acid (Sigma-Aldrich), sodium borohydride (NaBH_4_, Sigma-Aldrich), and silver nitrate (AgNO_3_, Sigma).

AuNRs were synthesized using a modified protocol from Burrows et al.^26^ First, a 0.1 M CTAB solution was prepared by suspending 0.3554 g of CTAB up to 9.75 mL of H_2_O. This solution was then stirred at 200 rpm and heated to 40 °C until the solution turned from white to clear (to dissolve the CTAB), at which point it was brought back down to 26 °C. While waiting, a fresh cold solution of 0.01 M NaBH_4_ was prepared by first diluting 0.1 M NaBH4 in 10 mL of H_2_O and keeping it on ice until its rapid use. Our seed solution was prepared by adding 250 μL of 0.01 M HAuCl_4_ into the CTAB solution and stirring for 1 min at 26 °C. After 1 min, 600 μL of the 0.01 M NaBH_4_ solution was added and left to stir for 1 h at 200 rpm at 26 °C. This resulted in a honey-colored solution. The growth step, which forms our nanorods, was performed by adding 500 μL of 0.01 M HAuCl_4_ to 9.5 mL of 0.1 M CTAB solution, followed by 100 μL of 0.01 M AgNO_3_, 55 μL of 0.01 M ascorbic acid, and 12 μL of the prepared seed solution. The mixture was gently stirred for 2 h and immediately collected for centrifugation at 11,000 rcf for 15 min. The supernatant was discarded, and the pellet was resuspended in the same volume of H_2_O as the supernatant was. This process was repeated two more times for a total of three washes. The final pellet was suspended in a quarter volume of the original volume to concentrate. The AuNRs were characterized using an M4 UV–visible (UV–vis) spectrophotometer (VWR International, Inc.) (Figure S2, left) and a Titan Themis 300 transmission electron microscope (TEM), Figure S2. Three UV–vis spectra were collected from a 1:10 dilution of AuNRs to H_2_O, as shown in Figure S2, right.

### 2.4 (Surface-Enhanced) Raman Spectroscopy

Validation samples were prepared by mixing 5 μL of AuNRs with 2.5 μL of artificial dye and depositing the mixture on a glass coverslip. For hair samples, 5 μL of AuNRs was applied per strand to coat the cuticle. Solutions were analyzed with the laser positioned at the center of the droplet, while hair was analyzed at the medulla and proximal regions. Five SER spectra were collected per dye or dye-developer solution, and 15 per hair sample (five spectra per strand, three strands). Additionally, dye application was performed once per product; however, spatial replication across strands and multiple acquisition points were used to capture intrasample heterogeneity. Data were acquired using a custom-built TE-2000U Nikon inverted confocal microscope with a 20× objective and a 785 nm solid-state CW laser (3 mW at sample, ND filter). Spectra (308–1952, ∼1.5 cm^–1^ resolution) were collected via the same objective, passed through a 10/90 beam splitter into an IsoPlane-320 spectrometer (600 groove/mm, 750 nm blaze), and detected with a PIX-400BR CCD. A Semrock LP03-785RS-25 long-pass filter blocked elastically scattered light. Acquisition times ranged from 1 to 20 s. Calibration with benzonitrile established a center wavelength of 784.7 nm. Instrument performance was monitored daily using benzonitrile Raman intensity at 1600 cm^–1^, yielding stability values of 185–536 counts·s^–1^ ·mW^–1^, with >100 counts consistently supporting reliable analyses.

### 2.5 Chemometric Analysis

All spectra were trimmed to the 450–1650 cm^–1^ range to reduce edge noise, baseline-corrected using the asymmetric least-squares (ASLS) algorithm (λ = 1 × 10^5^, *p* = 0.01), smoothed using a first-order Savitzky–Golay filter (window length = 7, polyorder = 1), and area-normalized prior to analysis, as displayed in the figures. This was done using Python 3.13 and the following libraries: NumPy, pandas, SciPy, pybaselines, and scikit-learn. A visual demonstration of our data before and after preprocessing is presented in Figure S3.

Furthermore, the following machine learning models were trialed in this study: logistic regression discriminant analysis (LRDA), partial least-squares discriminant analysis (PLSDA), random forest discriminant analysis (RFDA), extreme gradient boosting trees discriminant analysis (XGBDA), artificial neural networks discriminant analysis (ANNDA), and cosine similarity-based nearest neighbor classification (CSNNC). These models represent some of the most effective approaches reported in spectral classification literature.^27–29^ Each model represents a distinct approach to feature discrimination, ranging from probabilistic and projection-based methods to tree ensembles, deep learning, and distance-based classification. Model hyperparameters were tuned via a grid search during cross-validation using artificially generated dye mixtures. Table S3 provides a summary of each model’s optimized parameters and the associated Python libraries used for their implementation.

Additionally, accuracy, F1 scores, sensitivity, subset recall, and Matthews correlation coefficient (MCC) were calculated for each model, where applicable, using the Python libraries: scikit-learn, NumPy, and pandas. The F1 score represents the harmonic mean of precision and recall, providing a balanced metric that is especially useful when evaluating performance on imbalanced data sets. The MCC is a robust metric for binary classification that considers true and false positives and negatives, offering a more informative and balanced measure than accuracy alone, particularly under class imbalance. The same seeds were used in all models that generate random subsets of features (RFDA, XGBDA, and ANNDA). All predictions were made using a strict threshold of greater than 0.5, unless stated otherwise. All figures were generated using the matplotlib and seaborn Python libraries to ensure publication-quality visualizations of spectral trends, model performance, and class-wise comparisons.

## 3 Results and Discussion

### 3.1 First Impressions of Data

Constructing a robust database for colorant recognition requires first discerning the extent to which fluctuations in dye SERS signals depart from their authentic colorant signatures. The assumption that colorants are the primary contributors to the SERS signal of dyed hair has not been rigorously validated. To date, there appears to be no comprehensive study that directly compares the SERS spectra of individual colorants in isolation to those of commercial hair dyes containing them. This hypothesis was initially proposed in 2015, when Kurouski and Van Duyne observed that the SERS spectra of dye formulations in solution closely resembled those obtained from dyed hair, suggesting minimal structural or chemical alteration during application.^30^ A similar observation was made by Holman and colleagues in 2024 during investigations into the effects of soil erosion on hair dye retention using SERS.^14^

To further evaluate the contribution of colorants, we prepared equimolar mixtures of isolated dye components corresponding to known commercial formulations (Table S2). The resulting SER spectra demonstrated a high degree of spectral similarity to both commercial dyes and dyed hair samples (Figure 1). Importantly, the consistency in peak positions across synthetic mixtures, commercial products, and dyed hair indicates that the chemical identity of the colorants is largely preserved postapplication. While the signal intensity varied, these differences are likely due to proprietary concentration levels in commercial dyes rather than chemical alterations.

**Figure 1:**
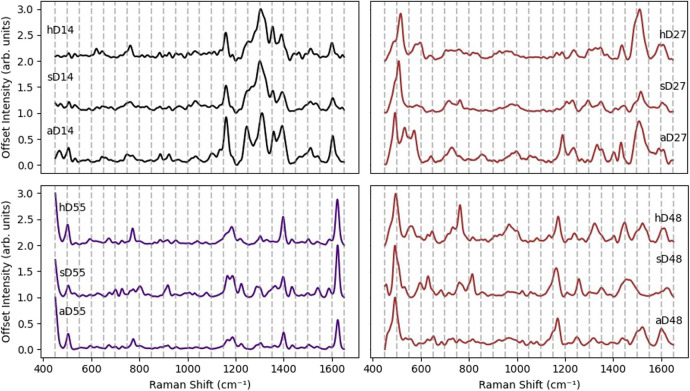
Examples for mean SER spectra and standard error (SE) of artificial dyes, real dye solutions, and dyed hair for (left) nonoxidative and (right) oxidative hair dyes. Dyed hair (hD#), commercial dye solutions (sD#), and their colorant-based artificial mixtures (aD#). Standard error bands are plotted but may be obscured due to high spectral similarity across replicates.

To further probe how colorants contribute to dye spectra, we evaluated whether their signals combine in a linear or nonlinear fashion. Using linear additive spectral mixing (LASM), we generated equal-weight combinations of individual colorants and compared them to the corresponding in-lab equimolar mixtures with cosine similarity (Figures 2 and 3). Because cosine similarity emphasizes the spectral shape rather than intensity, it provides a structure-focused measure of alignment. For nonoxidative dyes, such as aD14, the synthetic mixtures closely matched the experimental spectra (cosine similarity of 0.910), consistent with additive contributions of the individual colorants. By contrast, oxidative dyes showed weaker alignment; for example, aD27 yielded a cosine similarity of only 0.719, suggesting that chemical transformations alter their spectral profiles. Across all samples, nonoxidative dyes exhibited significantly higher cosine similarity (0.892 ± 0.075) in comparison to oxidative dyes (0.767 ± 0.038). A right-sided Welch’s *t-*test confirmed this difference (*t* = 4.873, *p* = 0.0004), reinforcing that nonoxidative dyes preserve colorant signatures more faithfully, while oxidative dyes undergo structural changes that complicate spectral interpretation.

**Figure 2:**
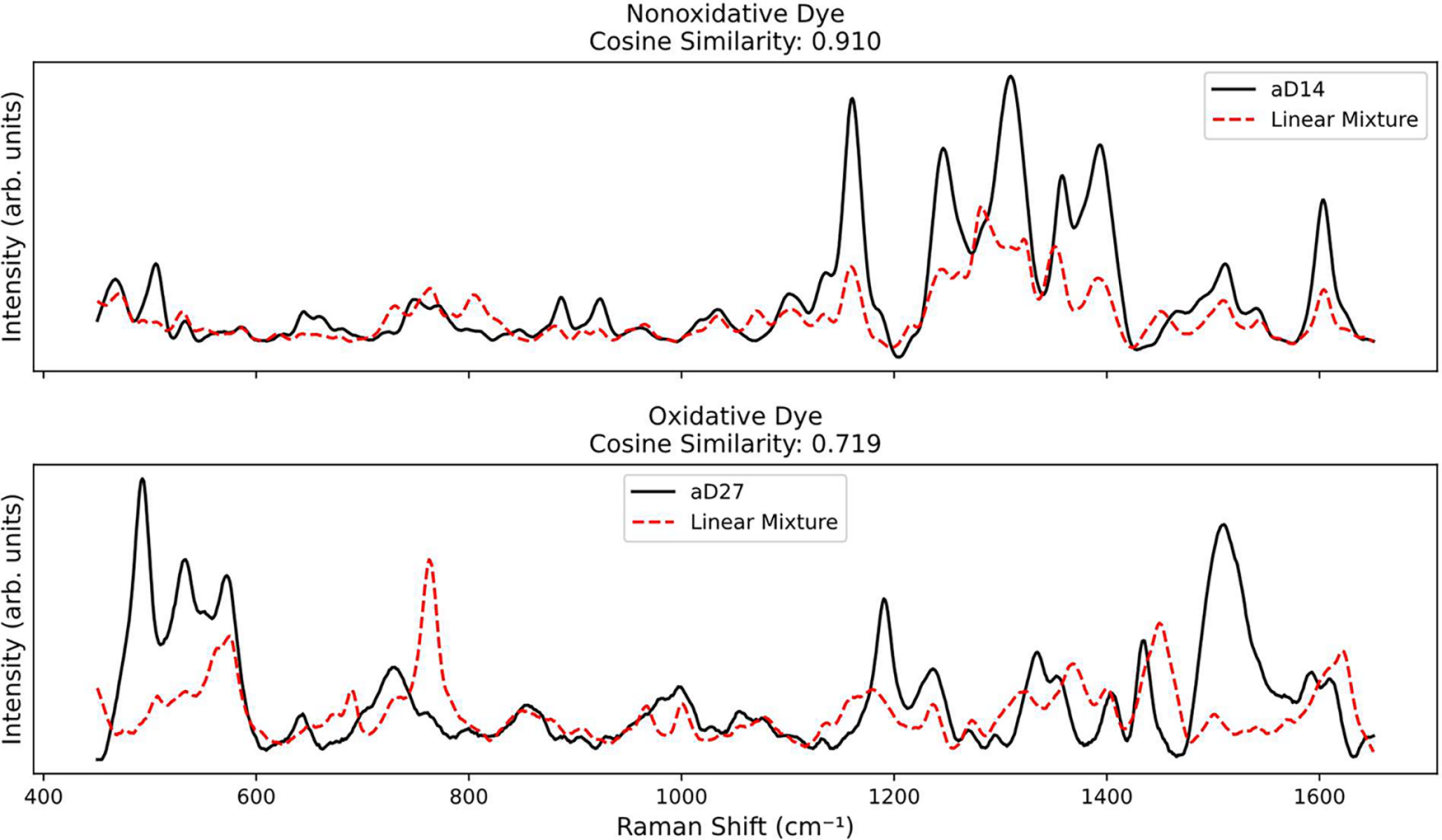
Comparison of SER spectra for two dyes, nonoxidative (top) and oxidative (bottom), and their equal-weighted colorant mixtures (Linear Mixture). Cosine similarity scores are reported in each panel. The high similarity observed for aD14 supports spectral additivity in nonoxidative dyes, while the lower similarity for aD27 suggests mixture-dependent chemical transformation or nonlinear mixing in oxidative dye systems.

**Figure 3:**
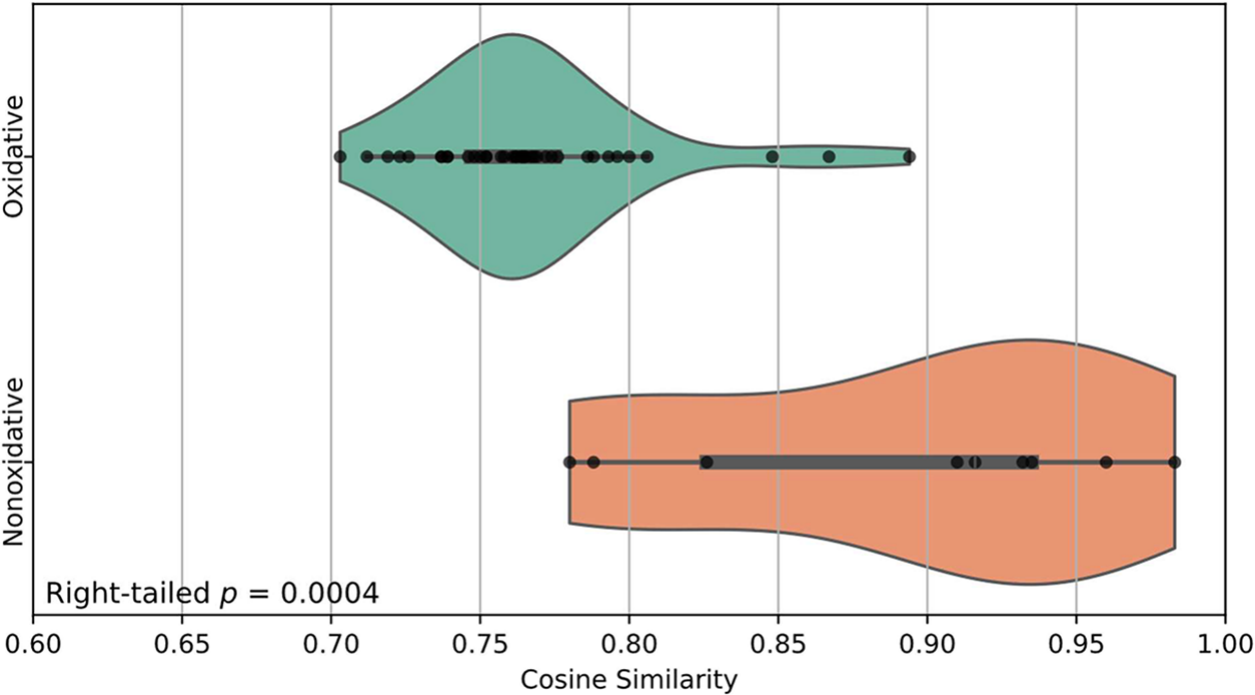
Comparison of cosine similarity scores between oxidative and nonoxidative hair dyes. Each distribution is visualized as a violin plot with overlaid data points. A one-tailed Welch’s *t-*test indicated that nonoxidative dyes had significantly higher cosine similarity scores than oxidative dyes (right-tailed *p* = 0.0004).

These results can be explained by the inherent reaction (un)­involved in nonoxidative vs oxidative dyes. As shown in Scheme S1, oxidative dyeing involves the in situ formation of novel chromophores through the oxidation of primary intermediates like *p*-phenylenediamine, followed by coupling with agents such as resorcinol. These reactions generate new vibrational features that are not present in the unreacted (couplers) or self-reacted (primary) components, making spectral reconstruction from individual precursors unsuitable. This key distinction underscores the need to treat oxidative and nonoxidative dye systems separately when developing spectral reference models for forensic classification.

### 3.2 Database Functionality

Accurate grouping of colorants, such as permanent vs direct or oxidative vs nonoxidative, is critical for meaningful analysis. Marketing claims often blur these distinctions, as in product D49, labeled “permanent” despite containing only Basic Red 51, a direct dye. In such cases, direct dyes act independently, producing SER signals that remain largely unaltered unless exposed to extreme oxidative conditions, which may degrade their Raman features. Direct dyes can also appear in oxidative formulations (e.g., D39), where their contribution depends on functional groups and possible binding. Outcomes may range from simple additive overlaps to complete spectral shifts from chemical interactions or degradation. Moreover, some colorants exhibit context-dependent behavior, functioning as direct dyes, couplers, or intermediates, depending on the oxidative environment, for example, 3-nitro-*p*-hydroxyethylaminophenol in products D10–D12 and in certain permanent dyes.

Given this complexity, DyeSPY adopts a tiered modeling framework (Scheme 1). Phase I first classifies spectra as oxidative or nonoxidative. In Phase II, nonoxidative samples are then analyzed using a predictive linear-overlap model, while oxidative samples are classified using a mixture-specific model to account for chemical transformations. Phase III subsequently predicts perceived color based on simplified product categories. Within this framework, products like D10–D12 and D49, although marketed as semipermanent or permanent, are treated as nonoxidative due to their underlying spectral behavior, ensuring that classification aligns with chemical reality rather than marketing labels.

**Scheme 1:**
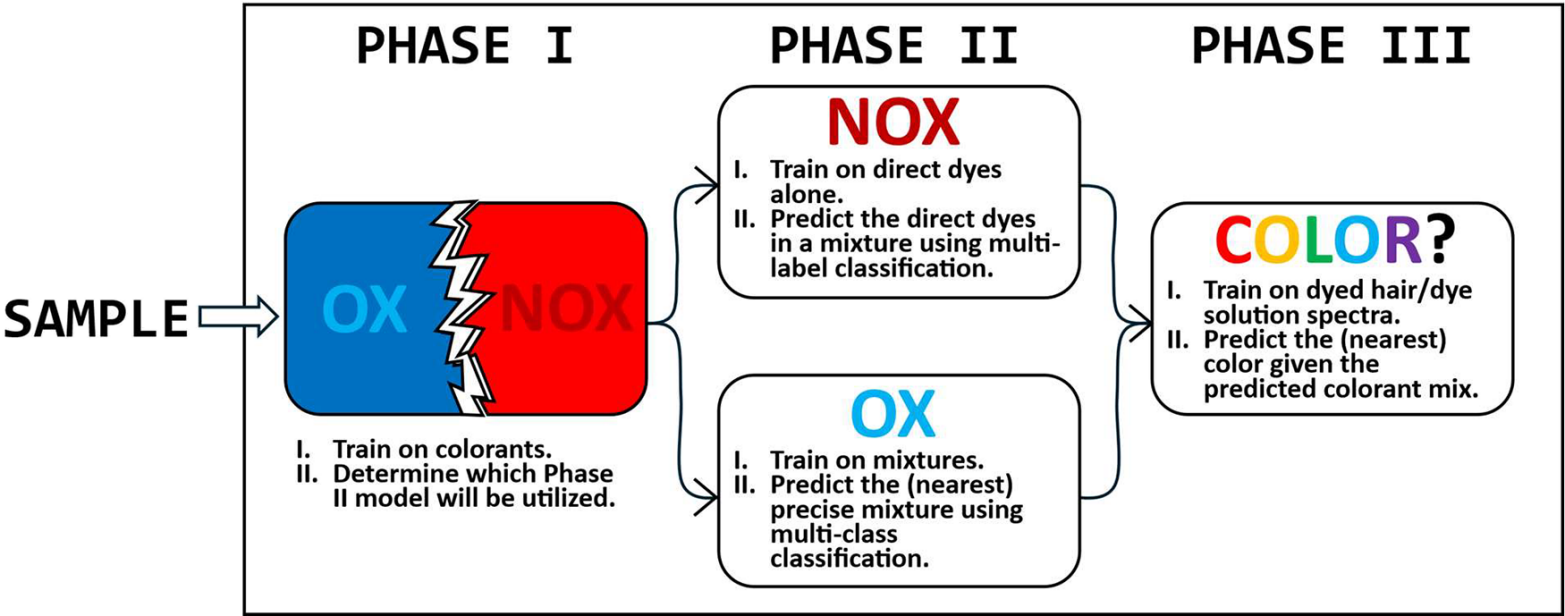
Preliminary Architecture of the DyeSPY Platform^a^

a Sample refers to a SER spectrum of a hair dye or dyed hair.

### 3.3 Phase I—Is the Sample Oxidative or Nonoxidative?

In this phase, multiple machine learning models (Tables 1 and S3) were trained on spectra from direct dyes (nonoxidative), primary intermediates (oxidative), and binary mixtures of primaries and couplers (oxidative), the latter included to capture the composite spectral signatures generated during oxidative dye formation (Scheme 1). Validation results (Table 2) showed consistently high performance across models in distinguishing oxidative from nonoxidative dyes. PLSDA and CSNNC achieved perfect classification on all metrics, demonstrating exceptional robustness in capturing subtle spectral differences. LRDA, RFDA, and XGBDA also performed extremely well, each yielding global F1 scores above 98% and high Matthews correlation coefficients (MCC), reflecting well-balanced predictions across classes. By contrast, ANNDA underperformed, reaching only 78% global F1 and accuracy; while it exhibited perfect sensitivity for oxidative dyes, it failed entirely to identify nonoxidative dyes, resulting in 0% sensitivity and MCC for that class.

After testing, interestingly, both linear models (LRDA and PLSDA) outperform the nonlinear models, namely, RFDA, XGBDA, and ANNDA, in terms of overall accuracy for dyed hair and dye solutions (Table 3). We note that the training data set contained more oxidative than nonoxidative spectra due to the inclusion of primary-coupler mixtures, which may have contributed to the asymmetric sensitivity observed. While PLSDA and CSNNC remained robust under this imbalance, the ANNDA architecture appeared more prone to overfitting toward the dominant oxidative class, resulting in 0% sensitivity for nonoxidative dyes. PLSDA stands out as the top-performing model overall, demonstrating the most balanced and accurate classification for both dyed hair and dye solutions. It achieved 98.56% accuracy on hair with 100% sensitivity for oxidative dyes on hair and 94.9% for nonoxidative dyes on hair, maintaining 88.33% accuracy on dye solutions with solid class sensitivities (90.7% oxidative, 82.35% nonoxidative). This balance of high accuracy and strong sensitivity for both classes suggests that PLSDA may be the most reliable and interpretable model for Phase 1.

**Table 3:** Testing Hyperparameter Tuned Phase I Models Using Dyed Hair (hD1–hD60) and Commercial Dye Solutions (sD1–sD60)

model	test set	accuracy, %	oxidative classification sensitivity, %	nonoxidative classification sensitivity, %	MCC
LRDA	hair	96.11	98.43	93.2	0.9089
LRDA	dye	90.0	95.35	76.47	0.7615
PLSDA	hair	98.56	100	94.9	0.9645
PLSDA	dye	88.33	90.7	82.35	0.7318
RFDA	hair	92.0	94.71	83.56	0.8035
RFDA	dye	83.33	89.18	63.77	0.5622
XGBDA	hair	92.44	100	73.33	0.8157
XGBDA	dye	89.33	97.67	68.24	0.7292
ANNDA	hair	71.67	100	0.0	0.0
ANNDA	dye	71.67	100	0.0	0.0
CSNNC	hair	97.78	100	92.16	0.9463
CSNNC	dye	87.33	100	55.29	0.6855

CSNNC, which was validated to perform well, achieved 97.78% accuracy on hair with perfect oxidative sensitivity (100%) and strong nonoxidative sensitivity (92.16%), but its performance on dyes alone dropped to 87.33% accuracy, again with perfect oxidative but reduced nonoxidative sensitivity (55.29%), suggesting generalization limitations. This difference likely reflects its underlying analytical basis: unlike PLSDA, a linear model that leverages spectral magnitude and variance-covariance structure, CSNNC operates on cosine similarity, emphasizing angular rather than intensity-based relationships. While this makes it robust to intensity variation, it can miss subtle spectral differences, particularly among nonoxidative dyes. These findings highlight the need to assess both overall accuracy and per-class sensitivity in chemically complex data sets like SERS spectra. For example, XGBDA and RFDA produced high accuracies (e.g., XGBDA at 92.44% on hair) but showed limited nonoxidative sensitivity (73.33% on hair; 68.24% on dyes), while ANNDA failed entirely to identify nonoxidative dyes (0% sensitivity) despite superficially strong overall accuracy driven by perfect oxidative classification. Such imbalances render these models unsuitable for the unbiased detection of both dye classes.

To further assess the robustness of PLSDA as the primary Phase I classifier, we examined its decision criteria by plotting the loading profiles for the first three latent variables (LV1–LV3) alongside a correlation heatmap of positive and negative loadings (Figure S4). Six dominant spectral regions consistently emerged across these latent variables, indicating stable discriminatory features rather than noise-driven separation. Across LV1–LV3, the ∼1600 cm^–1^ band carries substantial negative loadings, consistent with an aromatic ring C=C stretch.^31^ Interestingly, we also observe systematic sign changes in the 1060–1300 cm^–1^ region, which aligns with various (mainly para-) substituted aromatic constituents, including (primary) amine C–N stretching/deformation in the 1260–1300 region.^32,33^ The recurrence and sign-reversal of these loadings across LVs indicate that the model distinguishes dye pathways through consistent spectral contrasts rather than a single-feature dominance (i.e., similar magnitudes across LVs). This supports both the chemical plausibility and statistical stability of the Phase I classification.

In summary, the comparative evaluation of machine learning models in Phase I highlights PLSDA as the most balanced and robust performer, offering high accuracy and strong sensitivity for both oxidative and nonoxidative dye classes across hair and dye solution data sets. CSNNC follows closely, particularly excelling in hair analysis but showing some limitations in generalization to dye solutions. These findings underscore that high overall accuracy alone is insufficient for model selection in spectroscopic classification tasks. Sensitivity for each class must be carefully weighed, especially in domains such as forensic or diagnostic chemistry where missing a minority class can carry significant consequences.

### 3.4 Phase II—What Colorants Are in the Sample?

#### 3.4.1 Nonoxidative Colorant (Mixture) Classification

Initial efforts to classify nonoxidative dyes revealed substantial challenges across all model types, even after extensive hyperparameter optimization. The highly overlapping and subtle spectral features of direct dyes on hair limit the ability of conventional models to generalize effectively. To assess the feasibility, we applied a minimum performance threshold of either 70% subset recall (whether there is at least one correctly predicted colorant per sample) or a global F1 score. Approaches failing to meet this benchmark were excluded from further development. The models trialed and their outcomes are summarized in Table 4.

**Table 4:** Nonoxidative Classification Experimental Workflow^a^

initial model attempts	preliminary global F1 score/subset recall^a^	conclusion
trained model on pure direct dye spectra alone	<70% both	unsuccessful
trained model on spectra from laboratory-made nonoxidative dye mixtures	<70% both	unsuccessful
Improved and Final Approach		
generated synthetic spectra by linearly combining pure direct dye spectra with equal weighting; model trained on these synthetic mixtures performed significantly better at identifying the individual colorants	>70% subset recall (ANNDA)	successful

a Scores from testing on dyed hair spectra following cross-validation model selection.

The most effective strategy involved generating synthetic spectral mixtures through linear additive spectral mixing (LASM) of pure direct dye spectra with equal weighting (Figure 4), an approach that mimics the composite nature of dye formulations while minimizing variability from matrix effects. Models trained on these synthetic data sets consistently exceeded the 70% subset recall threshold, achieving a global F1 score of 0.8824 and a mean subset recall of 85.7%, with full compositional matches in most dyed hair samples (Table 5) and strong performance on commercial dye solutions. While overall results were robust, discrepancies arose in mixtures with overlapping colorants; for example, AX+BX+DX was correctly identified in hD59, but hD12 was misclassified as AX+GX. Such errors likely reflect nonlinear matrix effects, spectral overlap between structurally similar dyes, or concentration-dependent detectability limitations in the training set. These outcomes underscore the need to refine synthetic training strategies by incorporating more diverse and compositionally complex mixtures to further improve the generalizability.

**Figure 4:**
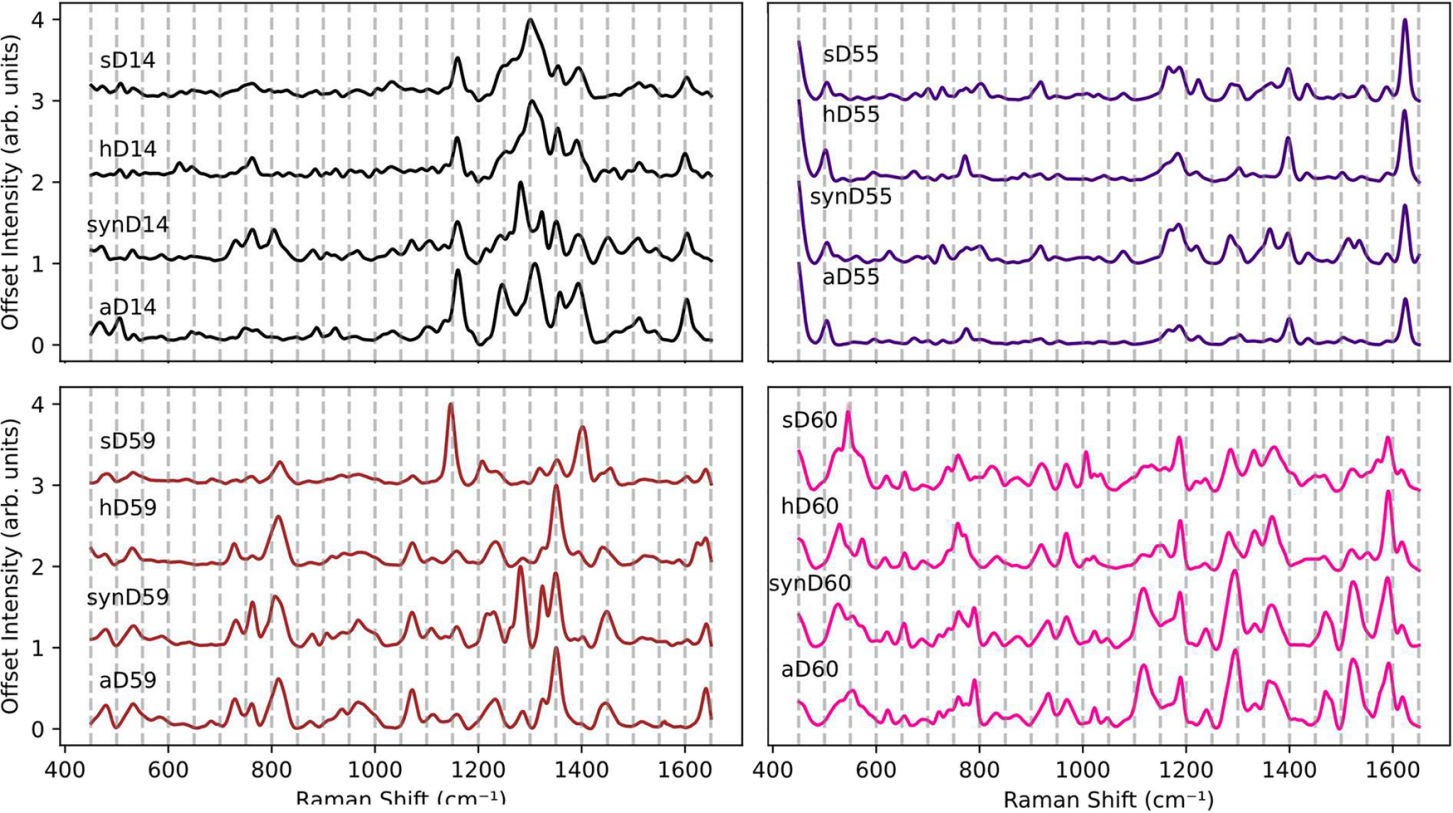
Mean and SE for SER spectra of LASM-generated mixtures (synD#), dyed hair (hD#), commercial dye solutions (sD#), and their colorant-based artificial mixtures (aD#). LASM-generated mixtures using averages from each pure colorant to generate its mixture and therefore have no SE. Standard error bands are plotted but may be obscured due to high spectral similarity across replicates.

**Table 5:** Performance Summary of Nonoxidative Dye Mixture Classification Using Synthetic Training Data Generated via Linear Additive Spectral Mixing (LASM) for Dyed Hair^a^

ESID(s)	number of spectra	true mixture	(majority) predicted mixture	proportion of samples predicted that, %	subset recall, %
hD10	15	(LL)+AX+DX+FX+GX	AX+DX+FX+GX	100	100
hD11	15	(LL)+CX+KX+JX	JX	100	100
hD12	15	(LL)+AX+BX+GX	AX+GX	100	100
hD13; hD49; hD52	45	HX	HX	100	100
hD14	15	AX+DX+FX+GX	AX+DX+FX+GX	100	100
hD50	15	IX	IX	100	100
hD52	15	DX	FX	100	0
hD53; hD57	30	NX	NX	100	100
hD54	15	FX+MX+OX	FX	100	100
hD55	15	JX+LX+MX+PX	JX+LX+MX+PX	100	100
hD56	15	AX+HX	AX+HX	100	100
hD58	15	KX		100	0
hD59	15	AX+BX+DX	AX+BX+DX	100	100
hD60	15	IX+NX	IX+NX	100	100

a Hyperparameters: layers = [512, 256], dropout = 0.3, optimizer = RMSprop, eta = 0.0005, batch size = 16, L2 = 0.001, L1 = 0.000001.

Equally notable were the results obtained for commercial dye solutions, as shown in Table S4. The global F1 score was 0.7647 for this model, with a mean subset recall of 71.4%. Despite these solutions presenting greater formulation variability and potential undisclosed ingredients, the model still maintained strong performance on several combinations. For example, complete identification was achieved for complex formulations such as (LL)+AX+DX+FX+GX and JX+LX+MX+PX, reaffirming the model’s robustness when applied to real-world product variability. However, a few samples, namely, sD52, sD56, sD58, and sD59, showed zero subset recall, indicating occasional limitations, often due to missing or misidentified minor components or labeling ambiguities within the commercial products.

In summary, the LASM-based synthetic data approach offers a powerful and practical solution for training machine learning models to identify colorant mixtures in forensically relevant dyed hair samples. By generating controlled, representative spectra from known pure dye components, this method overcomes challenges posed by experimental variability, matrix interference, and limited labeled mixture data. The high subset recall achieved across both laboratory-prepared and commercial dye formulations highlights the approach’s effectiveness for real-world forensic casework, where accurately determining the full composition of applied hair dyes is essential for evidentiary comparison, source attribution, and investigative reconstruction.

#### 3.4.2 Oxidative Colorant (Mixture) Classification

As discussed in Sections 3.1 and 3.2, LASM is ill-suited for oxidative colorant mixtures due to their reactive chemistry. Thus, the oxidative colorant classification took slightly different approaches, as described in Table 6.

**Table 6:** Oxidative Classification Experimental Workflow^a^

initial model attempts	preliminary global F1 score/subset recall^a^	conclusion
trained on pure primary intermediates alone and/or dual primary and coupler mixtures.	>70% subset recall (CSNNC)	modest success
trained model on spectra from laboratory-made oxidative dye mixtures	<70% both	unsuccessful
Improved and Final Approach		
trained model using 20% of commercially dyed hair and solution samples and tested on the remaining 80%	>70% both (ANNDA)	successful

a Scores from testing on dyed hair spectra following cross-validation model selection.

The initial modeling attempt, which used CSNNC to detect oxidative dye primaries, is summarized in Tables S5 and S6 for dyed hair and commercial dye solutions. Although the model achieved 83–90% accuracy in identifying at least one correct primary intermediate within the top two predicted labels, its discriminatory power was limited. Many permanent dyes share common primary structures (Table S2), reducing its forensic utility. To overcome this, we adopted an improved strategy using ANNDA trained on spectra from dyed hair and commercial products. The model was trained on 20% of the data set and tested on the remaining 80% to better simulate forensic deployment conditions, where reference spectra are limited relative to casework samples. This intentional reversal of the conventional 70–30 or 80–20 split serves to stress-test generalizability by maximizing exposure to unseen spectra and, as supported by statistical learning theory,^34,35^ reduces the variance of the generalization error estimate for a stricter assessment of out-of-sample robustness. This design choice prioritized failure-case detection over optimized in-sample fitting, with the resulting performance summarized in Tables 7 and S7.

**Table 7:** Performance Summary of Oxidative Dye Mixture Classification Using 20–80 Train-Test Partitioning of Dyed Hair SER Spectra^a^

ESID(s)	number of spectra	true mixture	(majority) predicted mixture	proportion of samples predicted that, %	subset recall, %
hD1	12	A+KK	A+KK	100	100
hD2, hD33	24	D+AA+DD+FF+PP	D+AA+DD+FF+PP	100	100
hD3, hD9	24	C+D+E+DD+HH+PP	C+D+E+DD+HH+PP	100	100
hD4	12	D+E+RR	D+E+RR	100	100
hD5	12	E+TT	E+TT	100	100
hD6	12	A+KK+NN	A+KK+NN	100	100
hD7	12	D+E+AA+FF+PP	D+E+AA+FF+PP	100	100
hD8	12	D+PP	D+PP	100	100
hD15	12	D+AA+DD+FF+NN+OO+UU	D+AA+DD+FF+NN+OO+UU	100	100
hD16	12	D+DD+GG+NN+OO+QQ	D+DD+GG+NN+OO+QQ	100	100
hD17	12	D+AA+OO+PP+TT	D+AA+OO+PP+TT	100	100
hD18	12	D+AA+FF+TT	D+AA+FF+TT	100	100
hD19	12	D+FF+OO+QQ+TT	D+FF+OO+QQ+TT	100	100
hD20	12	D+DD+UU	D+DD+UU	100	100
hD21	12	A+B+C+DD+FF+NN+OO	A+B+C+DD+FF+NN+OO	100	100
hD22	12	A+AA+BB+DD+NN	A+AA+BB+DD+NN	100	100
hD23	12	A+B+C+AA+DD+FF+NN	A+B+C+AA+DD+FF+NN	100	100
hD24	12	B+AA+DD+OO+PP+JJ	B+AA+DD+OO+PP+JJ	100	100
hD25	12	A+D+AA+DD+OO+PP	A+D+AA+DD+OO+PP	100	100
hD26	12	A+DD+HH	A+DD+HH	100	100
hD27	12	A+DD+FF+HH	A+DD+FF+HH	100	100
hD28	12	A+E+DD+FF+HH	A+E+DD+FF+HH	100	100
hD29	12	D+AA+DD+OO+PP	D+AA+DD+OO+PP	100	100
hD30	12	D+DD+FF+GG+OO+QQ	D+DD+FF+GG+OO+QQ	100	100
hD31	12	D+AA+DD+FF+OO+QQ	D+AA+DD+FF+OO+QQ	100	100
hD32	12	D+AA+DD+FF+OO	D+AA+DD+FF+OO	100	100
hD34	12	C+D+DD+OO+PP+IP	C+D+DD+OO+PP+IP	100	100
hD35	12	C+D+DD+HH+IP	C+D+DD+HH+IP	100	100
hD36, hD38	24	D+E+AA+OO+IP	D+E+AA+OO+IP	100	100
hD37	12	E+AA+HH+IP	E+AA+HH+IP	100	100
hD39	12	B+C+EE+FF+HH+OO+UU+DX	B+C+EE+FF+HH+OO+UU+DX	100	100
hD40	12	A+D+EE	A+D+EE	100	100
hD41	12	B+C+AA+NN+OO	B+C+AA+NN+OO	100	100
hD42	12	A+B+AA+DD+PP+UU	A+B+AA+DD+PP+UU	100	100
hD43	12	A+B+C+AA+EE+UU	A+B+C+AA+EE+UU	100	100
hD44	12	B+C+EE+FF+OO+PP+UU	B+C+EE+FF+OO+PP+UU	100	100
hD45	12	B+C+D+DD	B+C+D+DD	100	100
hD46	12	B+C+AA+DD	B+C+AA+DD	100	100
hD47	12	B+C+DD+FF	B+C+DD+FF	100	100
hD48	12	B+C+NN+OO	B+C+NN+OO	100	100
hNRs	12	NRs	NRs	100	100

a Hyperparameters: layers = [1024, 512], dropout = 0, optimizer = RMSprop, eta = 0.001, batch size = 32, L1 = 0, L2 = 0.

For dyed hair samples (Table 9), the model achieved perfect classification for all tested combinations with a global F1 score of 1.000 and a mean subset recall of 100%. Notably, even highly complex mixtures containing six to eight colorants, such as hD15 (D; AA; DD; FF; NN; OO; UU) and hD39 (B; C; EE; FF; HH; OO; UU; DX), were correctly predicted in full. These findings underscore the model’s ability to distinguish nuanced spectral profiles when trained on authentic chemical interactions embedded within the dyed substrate.

The classification performance for commercial dye solutions was similarly robust, although with slightly more variability (Table S7). The model maintained perfect subset recall and majority prediction accuracy for nearly all samples, with a global F1 score of 0.9821 and a mean subset recall of 98.5%. The only exceptions were mixtures such as hD3/hD9, where the true label was (C; D; E; DD; HH; PP) and the model returned (A; D; AA; DD; OO; PP). While subset recall remained at 100%, this discrepancy highlights the potential for confounding among structurally or spectrally similar components, particularly where colorants may share overlapping absorption or scattering features. The improved classification accuracy not only validates the use of data-driven spectral models in forensic hair dye analysis but also reinforces the importance of incorporating real oxidative behavior into the training process.

Together, these findings support the implementation of hybrid modeling pipelines: LASM-based synthetic spectra for stable nonoxidative mixtures and empirical training on oxidized formulations for chemically reactive permanent dyes. When applied to forensic investigations, this approach maximizes both coverage and specificity, enhancing the evidentiary value of SER analysis in dyed hair trace evidence comparisons.

### 3.5 Phase III—What Is the Original Color?

Color is one of the most distinguishing features of dyed hair, making accurate characterization essential for forensic comparisons.^36^ While traditional visual or spectroscopic methods provide useful insights, subtle color differences arising from dye formulation, manufacturing variations, and environmental degradation can complicate a direct comparison. Compounding this issue, commercial hair dye products are often labeled with highly stylized names such as “Wrath,” “Dark Sand,” or “Sour Candy” (see Table S2), which convey little about the actual appearance of the dye. For example, few would intuit that “Electric Paradise” refers to a bright pink hue. Such names not only hinder objective classification but also hinder witness-driven investigations. Asking a witness if they saw someone with “Sour Candy” hair could result in confusion or misidentification.

To address limitations in communication among forensic analysts, investigators, and laypersons, we implemented a simplified colorimetric labeling scheme based on the visible appearance of dyes on hair (Table S8). This system prioritizes clarity and consistency over brand-specific terminology. Research by Emery and Webster shows that while basic color categories are perceived consistently, nuanced hues vary widely, supporting the use of standardized labels in forensic contexts.^37^ Simplified classification reduces ambiguity in casework, where subjective terms like “auburn” or “golden chestnut” can create confusion in evidence comparison or testimony. By grounding labels in perceptual consensus rather than proprietary branding, our approach aligns with SWGMAT guidelines, “[bridging] analytical precision with visual intelligibility.”^36^

Since our goal was to infer hair dye identity from spectral data, we adopted a machine learning approach that matches each sample to the most spectrally similar dye class and then assigns the corresponding simplified color. Given the likelihood of encountering degraded or low-quality evidence in the casework, we avoided models dependent on spectral magnitude, which can be affected by SERS enhancement variability, colorant concentration variation, and sample contamination. Instead, we trained a CSNNC that emphasizes spectral shape over intensity, improving robustness in trace or compromised forensic samples.

To ensure contextual accuracy and reduce bias from matrix-specific spectral variability, we trained separate CSNNC models for dyed hair samples and commercial dye solutions. Each model was trained using 20% of the spectra from its respective data set and evaluated on the remaining 80%. This split preserved spectral heterogeneity in the test set and emphasized the model’s ability to generalize across samples (Table 8).

**Table 8:** CSNNC Metric Results for the Color Characterization of True and Shuffled Mixture IDs

train/test set	Youden’s J threshold	min similarity to true mixture	max similarity to shuffled (false) mixture	accuracy, %	false positive rate, %	false negative rate, %
hair	0.9065	0.8601	0.9996	97.92	1.39	2.78
dye	0.9469	0.6864	0.9903	97.5	3.33	1.67

Additionally, to assess the impact of incorrect mixture identification on the downstream color prediction, we performed a controlled label-shuffling experiment. Specifically, mixture IDs were randomly reassigned to all samples within each model to simulate misclassification. Cosine similarity scores between true and false pairings were then compared, and we identified the optimal similarity threshold, known as Youden’s J statistic, that maximized the difference between the true positive rate and false positive rate (Table 8). This threshold served as a decision boundary for distinguishing genuine matches from spectral impostors. Cosine similarity plots illustrating the separation between true and false pairings are presented in Figure S5.

Using Youden’s J statistic to optimize the decision threshold, the models successfully separated true mixture assignments from shuffled (false) IDs based on cosine similarity. Specifically, both models achieved high classification accuracy for color characterization: 97.92% for dyed hair and 97.5% for dye solutions. The optimal threshold was 0.9065 for hair and 0.9469 for dye solutions. Importantly, the maximum similarity observed for shuffled IDs exceeded 0.99 in both data sets, demonstrating the necessity of a carefully defined threshold to prevent false positives. Despite this challenge, the models maintained low false positive and false negative rates, with hair samples showing particularly strong separation (FPR: 1.39%, FNR: 2.78%).

These results support the utility of CSNNC as a robust method for classifying the color of dyed hair based on predicted colorant mixtures, particularly under conditions where absolute intensity is unreliable. The method proved to be effective for both dyed hair and dye solutions, highlighting its broad potential for forensic application.

### 3.6 Database Accessibility and Appearance

We developed an application programming interface (API) to enable the streamlined classification of SER spectra obtained from dyed hair or commercial dye solutions. The API serves as the operational backbone of the DyeSPY pipeline, handling data preprocessing, pathway classification (oxidative vs nonoxidative), colorant mixture identification, and perceptual color prediction in a fully automated manner.

The API is modularized into three main phases, each linked to independent scripts and pretrained (and hyperparameter tuned) models, and is designed to ingest unknown samples and return interpretable forensic results. Figure 5 outlines the structure of the DyeSPY pipeline, including both dyed hair and commercial dye modules, with explicit mapping between phases, scripts, and trained model assets. This can be used as a reference guide to understand the backbone of each script that was modularized in the run_pipeline.py script for both “DyedHairModules” and “CommercialDyeModules” characterization folders.

**Figure 5:**
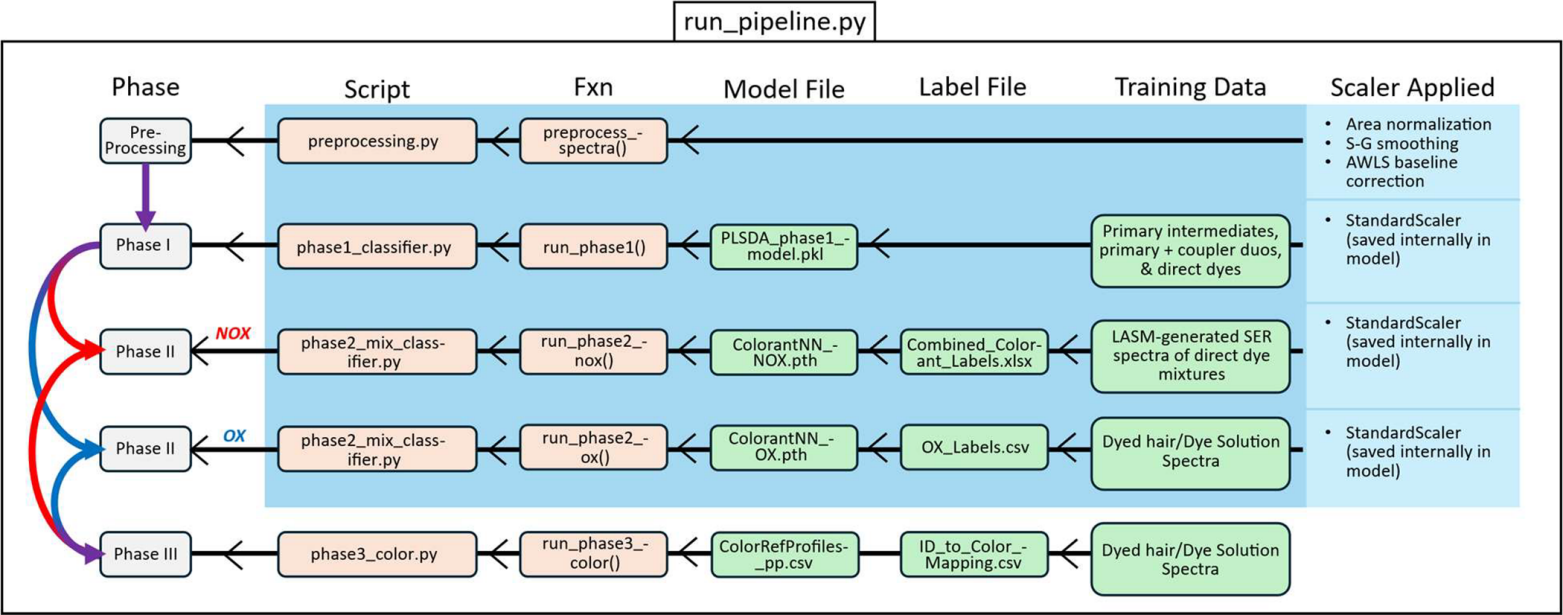
DyeSPY pipeline component summary for both dyed hair and commercial dye characterization modules.

Figure 6 provides a test example of how an unknown forensic sample, along with positive and negative controls, would be processed through the API, demonstrating end-to-end classification and decision support. A user can replace their .csv file with the area where “DyeSPY_test.csv” is currently occupied in the run_pipeline.py script. Their .csv file shall be formatted to include their lab-specific ID number (“ID”) and Raman shifts that must include 450–1650 cm^–1^. Running the run_pipeline.py script in the terminal displays the process and predictions for each individual spectrum in the test file (Figure 6, right). Figure 7 shows the final lines of the script in run_pipeline.py that save the summary results and how those summary results are formatted.

**Figure 6:**
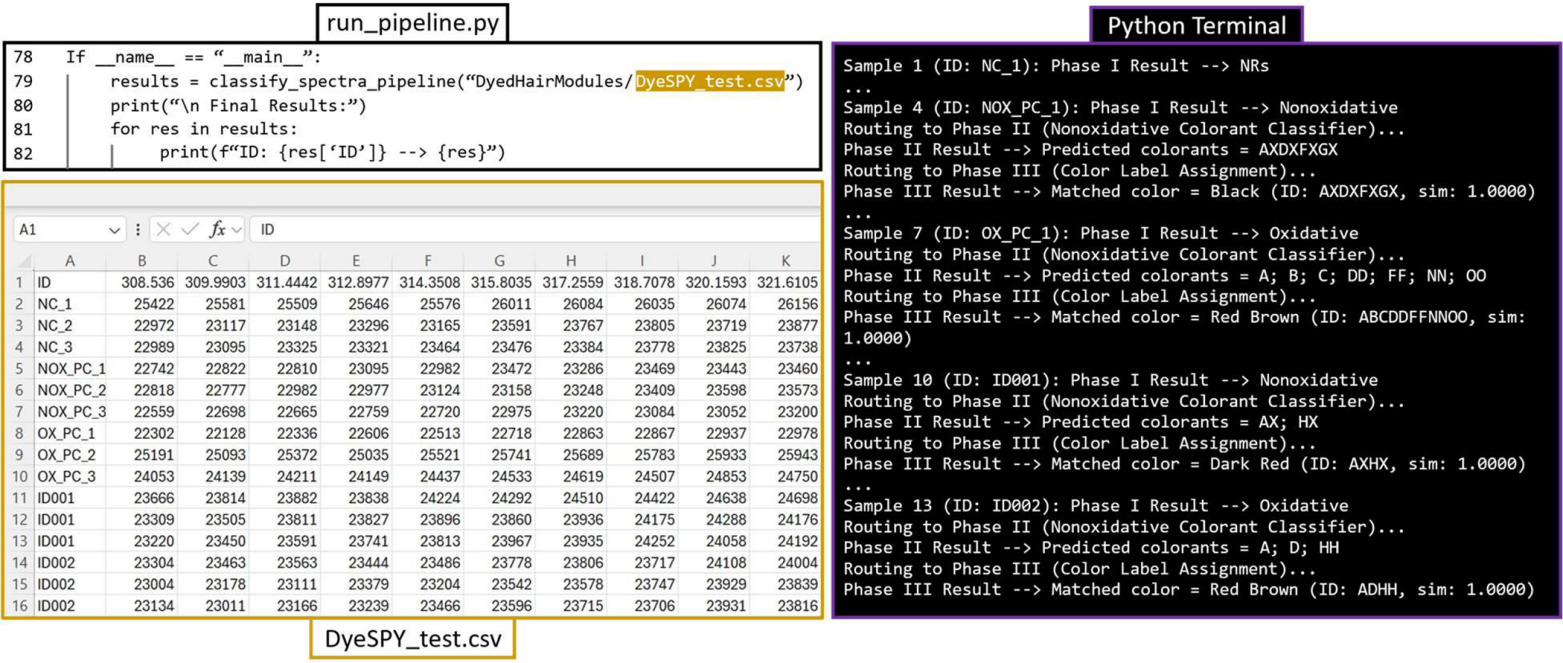
Example of how users’ samples would be processed using our DyedHairModules pipeline. A user would change the file name on line 79 of run_pipeline.py, where DyeSPY_test.csv ensures that the file is formatted the same. NC: negative control (AuNRs on hair); NOX_PC: nonoxidative positive control (hD14); OX_PC: oxidative positive control (hD21); ID001 = hD56; ID002 = hD26. A number of spectra per sample were reduced to 3 for figure purposes and are recommended to still follow a minimum of 5 spectra per hair strand, with 3 hair strands per sample (minimum of 15 spectra).

**Figure 7:**
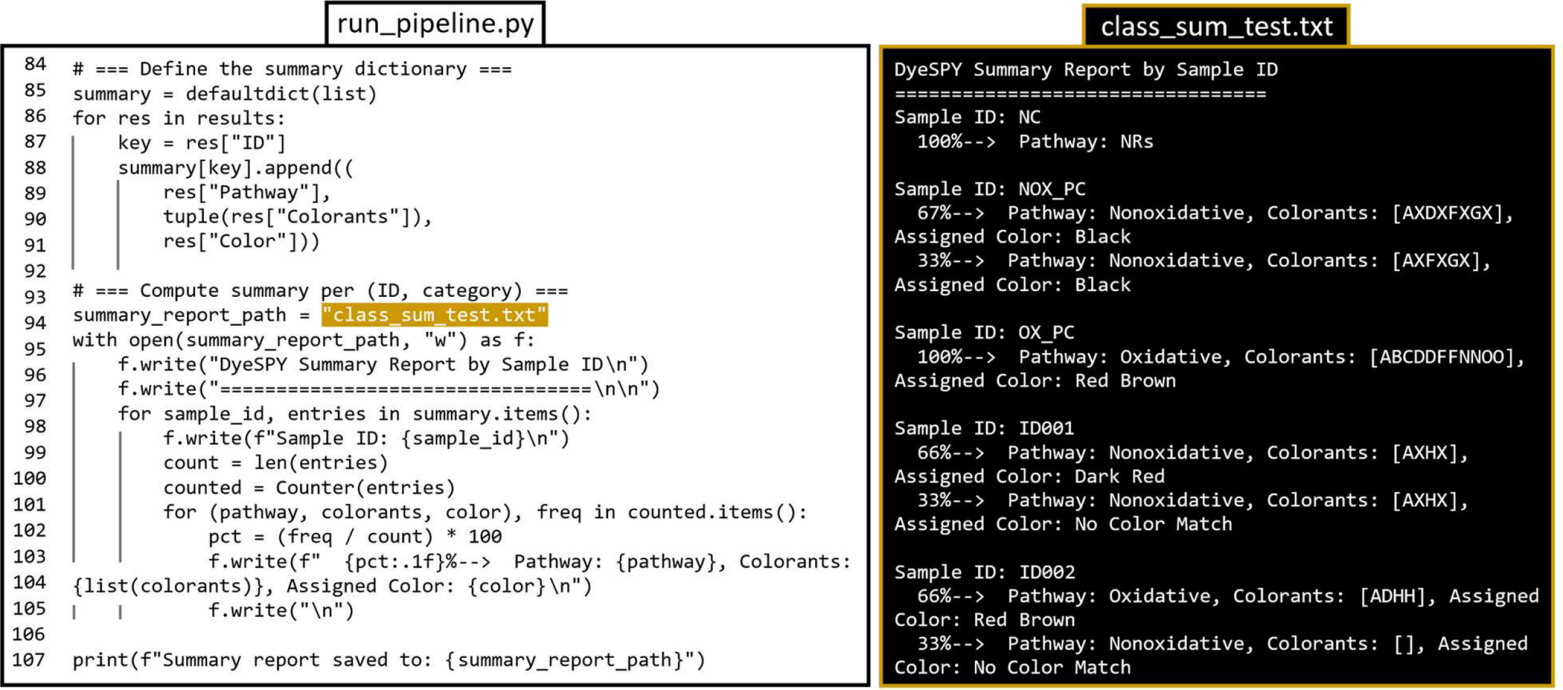
Continued script and example.txt file for summary results using DyeSPY and the DyeSPY_test.csv data set. A user would change the file name on line 94 of run_pipeline.py, where class_sum_test.txt is used to save a distinct summary file for records. The mispredictions shown in class_sum_test.txt are to give readers an idea of how DyeSPY displays summary results for samples with spectra that take different paths and do not represent the true results of samples used in this study.

To ensure accessibility, reproducibility, and cross-platform deployment, the API was containerized using Docker. This allows any user to build and run the complete pipeline with simple commands in a virtualized environment that includes all required Python packages (e.g., PyTorch, pandas, scikit-learn, and pybaselines). All scripts, model weights, scalers, and label files are version-controlled in a private GitHub repository (available upon request and approval), allowing researchers to (i) download and modify specific modules, (ii) push updates for collaborative improvement, and (iii) maintain reproducibility across forensic laboratories.

Additionally, the use of Docker containerization enables users to execute the DyeSPY pipeline securely and efficiently on their local machines without relying on external servers or cloud-based resources. This local execution environment allows all input files, intermediate results, and final outputs to be processed and stored directly on the user’s system.^38^ Such architecture is especially critical in forensic contexts, where the integrity, confidentiality, and chain-of-custody of evidentiary data must be rigorously maintained.^38^ By isolating the application in a controlled container and avoiding unnecessary network exposure, Docker ensures that sensitive spectral data and case-related metadata remain fully protected throughout the analysis process.

### 3.7 Cross-Study Validation

It is no secret that multiple groups following the same nanoparticle synthesis protocol will create various-sized AuNRs. These differences may be attributed to the intrinsic purity of the chemicals used as well as extrinsic factors such as the temperature of the room where the reactions are performed, type of glassware (flask vs beaker), instrumental error in stirring and heating, and so on.^39,40^ The question then becomes whether differently obtained AuNRs (and slightly different acquisition parameters) will elicit the same accuracies for the samples reported here by using our database.

Fortunately, our group has accumulated a large library of dyed hair spectra over a large number of experiments,^13–17,13–17,41–44^ each with their own synthesized AuNRs, across several protocols. To sufficiently answer questions on our databases’ performance against externally (to this study) obtained SER spectra of dyed hair, we selected only two studies: the spectral library from Higgins and Kurouski^42^ and Holman and Kurouski (Figure 8).^14^ Higgins and Kurouski demonstrated that through machine learning, SER spectra could be used to identify over 30 dyes on hair with 97% accuracy. Importantly, 21 of the hair dyes used in that study are also used in our database. On the other hand, Holman and Kurouski probed the effects of photodegradation of hair dyes over time and found that all hair dyes could be differentiated with over 90% sensitivity over a 10-week period. Of the four hair dyes tested in that study, three appear in this library (D1, D7, and D14) (Figure 8). Hair dyes that were exclusively used in the other studies were given ESIDs, as can be found in Table S9.

**Figure 8:**
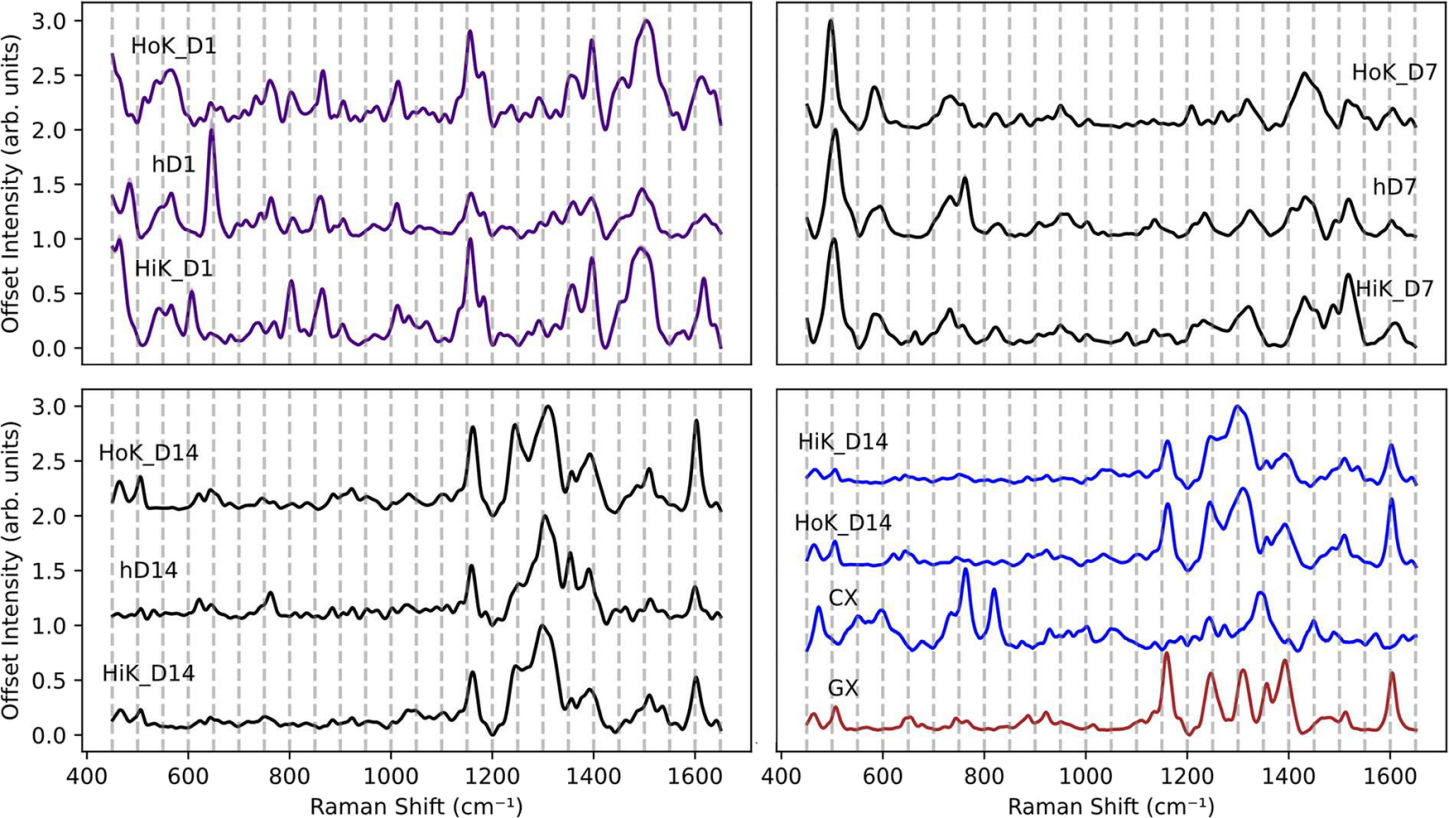
Examples for mean SER spectra and SE of dyed hair found in Higgins and Kurouski^42^ (HiK) and Holman and Kurouski^13^ (HoK) (before sun exposure) that are shared in this study (D1, D7, and D14) and one not shared (hD001) along with its misclassified colorants (GX and CX). Standard error bands are plotted but may be obscured due to high spectral similarity across replicates.

Using the DyedHairModules pipeline, we found that models trained on internal spectra generalized reasonably well to external data sets, though performance varied across phases (Table 9). In the Higgins and Kurouski data set, Phase I accuracy remained stable at 71.4%, demonstrating the robustness of the oxidative vs nonoxidative classification across different AuNR batches and preparation methods. Phase II performance was lower (62.9%), with frequent partial or incorrect mixture predictions, although samples such as D7 and D23 still showed high colorant precision. Phase III accuracy reached 66.7%, reflecting its dependence on Phase II fidelity; incorrect Phase II predictions typically defaulted to “No Color Match”, an appropriate conservative outcome. When colorants were correctly identified, perceptual color was reliably assigned (e.g., D7, D9, and D23), though misclassifications such as the reversed D10 and D14 highlight the need for standardized AuNR protocols and suggest that classifying color within ranges may improve interpretability. Overall, these results underscore the pipeline’s stability in Phase I, its sensitivity to spectral fidelity in Phase II, and the cascading impact on Phase III outcomes.

**Table 9:** Cross-Study Validation of DyeSPY Using Raw SER Spectra of Dyed Hair Groups That Were Included in This Study but Produced from Higgins and Kurouski,^42^ Utilizing the DyedHairModules Pipeline^a^

ESID	number of spectra	Phase I prediction	Phase I actual	Phase II prediction	Phase II actual	Phase III prediction	Phase III actual
D1	50	oxidative (82%)^b^	oxidative	A+KK+NN (64%)	A+KK (18%)	no color match (78%)	dark blue (0%)
D2	50	nonoxidative (100%)	oxidative	CX (100%)	D+AA+DD+FF+PP	no color match (100%)	yellow brown
D3	50	oxidative (62%)	oxidative	IC	C+D+E+DD+HH+PP (8%)	no color match (100%)	red brown
D4	50	oxidative (100%)	oxidative	D+E+RR (100%)	D+E+RR	no color match (100%)	purple
D5	50	nonoxidative (100%)	oxidative	AX	E+TT	no color match (100%)	dark red
D7	50	oxidative (100%)	oxidative	D+E+AA+FF+PP (60%)	D+E+AA+FF+PP	black (60%)	black
D9	50	nonoxidative (100%)	oxidative	AX+GX (98%)	C+D+E+DD+HH+PP	red brown (98%)	red brown
D10	50	nonoxidative (100%)	nonoxidative	AX+DX+FX+GX (100%)	LL+AX+DX+FX+GX	black (80%)	brown (20%)
D11	50	nonoxidative (100%)	nonoxidative	JX (82%)	LL+CX+KX+JX	pink (100%)	pink
D14	50	nonoxidative (100%)	nonoxidative	AX+DX+FX+GX (78%)	AX+DX+FX+GX	brown (100%)	black
D15	50	oxidative (100%)	oxidative	IC	D+AA+DD+FF+NN+OO+UU (0%)	no color match (70%)	brown
D16	50	nonoxidative (100%)	oxidative	No Match (62%)	D+DD+GG+NN+OO+QQ (0%)	no color match (100%)	red brown
D17	50	oxidative (100%)	oxidative	D+E+AA+FF+PP (68%)	D+AA+OO+PP+TT	IC	black (48%)
D18	50	oxidative (100%)	oxidative	D+DD+GG+NN+OO+QQ (74%)	D+AA+FF+TT (18%)	no color match (68%)	brown (16%)
D19	50	nonoxidative (90%)	oxidative (10%)	No Match (84%)	D+FF+OO+QQ+TT (0%)	no color match (100%)	brown
D20	50	oxidative (100%)	oxidative	D+AA+OO+PP+TT (52%)	D+DD+UU (0%)	black (68%)	black
D21	50	oxidative (100%)	oxidative	B+AA+DD+OO+PP+JJ (72%)	A+B+C+DD+FF+NN+OO (0%)	no color match (52%)	red brown
D22	50	oxidative (100%)	oxidative	B+C+AA+DD (96%)	A+AA+BB+DD+NN	no color match (100%)	black
D23	50	oxidative (80%)	oxidative	B+C+AA+DD (80%)	A+B+C+AA+DD+FF+NN	light brown (92%)	light brown
D24	50	nonoxidative (74%)	oxidative (26%)	No Match (60%)	B+AA+DD+OO+PP+JJ (14%)	dark purple (54%)	dark purple
D25	50	oxidative (100%)	oxidative	D+AA+OO+PP+TT (76%)	A+D+AA+DD+OO+PP (24%)	black (76%)	dark blue (24%)
total samples	total spectra	Phase I accuracy^c^	Phase II accuracy^c^ ^d^ ^e^	Phase II nonoxidative subset recall^c^ ^e^	Phase II oxidative subset recall^c^ ^e^	Phase II subset recall^c^ ^e^	Phase III accuracy^c^ ^e^ ^f^
21	1050	71.4%	62.9%	100%	83.3%	86.7%	66.7%

a IC: inconclusive for predictions with ≤50% spectral support.

b Percentages indicate the proportion of spectra predicted in that category.

c Calculated at the sample level; spectral percentages do not affect these values. IC is treated as incorrect.

d Phase II accuracy = (correct colorants ÷ total predicted colorants), averaged across samples.

e Phase II and III metrics include only samples correctly classified in Phase I.

f Phase III accuracy counts “No Color Match” as correct for incorrect colorant predictions, but incorrect when true colorants are correctly predicted.

As detailed in Table 10, spectra from Holman and Kurouski further tested DyeSPY’s resilience under photodegradation conditions. For dyes D1 and D14, which remained chemically stable over the first 7 weeks, Phases I and II predictions stayed consistent (100 and 78.6% accuracy, respectively), though Phase III again struggled to resolve colors due to subtle shifts in spectral features (23.8% accuracy). Interestingly, for D7, Phase II predictions became increasingly incongruent with time, shifting from correct predictions (Week 0) to more unrelated dye combinations. This may suggest that prolonged UV exposure can chemically transform dye residues into forms that mimic different colorant signatures in the Raman space. Despite these shifts, Phase I classification remained robust across all weeks (100%), supporting the use of this initial phase as a reliable decision point.

**Table 10:** Cross-Study Validation of DyeSPY Using Raw SER Spectra of Dyed Hair Groups That Were Included in This Study but Produced from Holman and Kurouski,^13^ Utilizing the DyedHairModules Pipeline^a^

ESID	number of spectra	weeks in the sun	Phase I prediction	Phase I actual	Phase II prediction	Phase II actual	Phase III prediction	Phase III actual
D1	50	0	oxidative (100%)^b^	oxidative	A+KK (100%)	A+KK	no color match (100%)	dark blue
	50	1	oxidative (100%)	oxidative	A+KK (100%)	A+KK	no color match (100%)	dark blue
	50	2	oxidative (100%)	oxidative	A+KK (60%)	A+KK	no color match (100%)	dark blue
	50	3	oxidative (78%)	oxidative	A+KK (78%)	A+KK	no color match (100%)	dark blue
	50	4	oxidative (100%)	oxidative	A+KK (100%)	A+KK	No Color Match (100%)	dark blue
	50	5	oxidative (100%)	oxidative	A+KK (74%)	A+KK	no color match (100%)	dark blue
	50	6	oxidative (100%)	oxidative	E+TT (66%)	A+KK (34%)	no color match (100%)	dark blue
	50	7	oxidative (100%)	oxidative	A+KK (68%)	A+KK	no color match (100%)	dark blue
	D1 samples	D1 spectra	D1 Phase I accuracy^c^		D1 Phase II accuracy^c^,^d^,^e^	D1 Phase II subset recall^c^,^e^	D1 Phase III accuracy^c^,^e^,^a^	
	8	400	100%		85.7%	85.7%	14.3%	
D7	50	0	oxidative (100%)	oxidative	D+E+AA+FF+PP (52%)	D+E+AA+FF+PP	IC	black (20%)
	50	1	oxidative (100%)	oxidative	IC	D+E+AA+FF+PP (0%)	IC	black (32%)
	50	2	oxidative (100%)	oxidative	D+AA+OO+PP+TT (70%)	D+E+AA+FF+PP (0%)	no color match (100%)	black
	50	3	oxidative (100%)	oxidative	A+D+AA+DD+OO+PP (72%)	D+E+AA+FF+PP (0%)	dark blue (100%)	black
	50	4	oxidative (100%)	oxidative	A+E+DD+FF+HH (64%)	D+E+AA+FF+PP (0%)	no color match (94%)	black (0%)
	50	5	oxidative (100%)	oxidative	A+D+AA+DD+OO+PP (100%)	D+E+AA+FF+PP	dark blue (100%)	black
	50	6	oxidative (100%)	oxidative	E+TT (74%)	D+E+AA+FF+PP (0%)	no color match (96%)	black (0%)
	50	7	oxidative (100%)	oxidative	E+TT (98%)	D+E+AA+FF+PP (0%)	no color match (100%)	black
	D7 samples	D7 spectra	D7 Phase I accuracy^c^		D7 Phase II accuracy^c^,^d^,^e^	D7 Phase II subset recall^c^,^e^	D7 Phase III accuracy^c^,^e^,^a^	
	8	400	100%		50%	85.7%	57.1%	
D14	50	0	nonoxidative (100%)	nonoxidative	AX+DX+FX+GX (80%)	AX+DX+FX+GX	brown (80%)	black (20%)
	50	1	nonoxidative (100%)	nonoxidative	AX+FX+GX (62%)	AX+DX+FX+GX (38%)	brown (100%)	black
	50	2	nonoxidative (100%)	nonoxidative	AX+DX+FX+GX (100%)	AX+DX+FX+GX	brown (100%)	black
	50	3	nonoxidative (100%)	nonoxidative	AX+DX+FX+GX (54%)	AX+DX+FX+GX	brown (80%)	black (20%)
	50	4	nonoxidative (100%)	nonoxidative	AX+DX+FX+GX (98%)	AX+DX+FX+GX	No Color Match (68%)	black (0%)
	50	5	nonoxidative (100%)	nonoxidative	FX (56%)	AX+DX+FX+GX (38%)	brown (74%)	black (0%)
	50	6	nonoxidative (100%)	nonoxidative	AX+DX+FX+GX (52%)	AX+DX+FX+GX	no color match (60%)	black (0%)
	50	7	nonoxidative (82%)	nonoxidative	DX+FX (54%)	AX+DX+FX+GX (6%)	no color match (100%)	black
	D14 samples	D14 spectra	D14 Phase I accuracy^c^		D14 Phase II accuracy^c^,^d^,^e^	D14 Phase II subset recall^c^,^e^	D14 Phase III accuracy^c^,^e^,^f^	
	8	400	100%		100%	100%	0%	
total samples	total spectra	Phase I accuracy^c^	Phase II accuracy^c^,^d^,^e^	Phase II nonoxidative subset recall^c^,^d^	Phase II oxidative subset recall^c^,^d^	Phase II subset recall^c^,^e^	Phase III accuracy	
24	1200	100%	78.6%	100%	85.7%	90.5%	23.8%	

a IC: inconclusive for predictions with ≤50% spectral support.

b Percentages indicate the proportion of spectra predicted in that category.

c Calculated at the sample level; spectral percentages do not affect these values. IC is treated as incorrect.

d Phase II accuracy = (correct colorants ÷ total predicted colorants), averaged across samples.

e Phase II and III metrics include only samples correctly classified in Phase I.

f Phase III accuracy counts “No Color Match” as correct for incorrect colorant predictions, but incorrect when true colorants are correctly predicted.

Table S12 further underscores the limits of spectral generalizability under extreme degradation conditions (weeks 8–10). For clarity, Holman and Kurouski stated that SER signal of dyes was visually unrecognizable by week 8.^13^ So, unsurprisingly, in all cases, Phase II accuracy dropped substantially, with many dyes falsely predicted as E+TT or FX, and Phase III predominantly failing to return correct colors. This collapse in specificity is consistent with the visual fading and broadening of SER bands seen in that study and emphasizes the importance of the spectral quality and dye stability in forensic classification contexts. Interestingly, D14 returned to highly accurate Phases I and II predictions at weeks 9 and 10. This observation supports that dye degradation is not uniformly progressive and may depend on the unique photochemical behavior of individual colorants.^45,46^

Although Phase II oxidative modeling underperformed relative to the nonoxidative classifier in overall accuracy, subset recall remained high across both validation studies. This indicates that DyeSPY often identifies a meaningful portion of the true dye mixture even when full matches are not achieved. A reclassification strategy that accounts for partial overlap may, therefore, be more appropriate for complex oxidative formulations. For instance, in sample D7, the model correctly predicted all five colorants (D+E+AA+FF+PP), while in sample D17, three of five predictions overlapped with the true mixture, still providing useful evidentiary value. These findings suggest that subset recall offers a more informative metric than exact-match accuracy and that forensic interpretation should prioritize component overlap when evaluating oxidative dye predictions.

External validation with dyes not represented in the DyeSPY training library (e.g., HC Blue No. 15, Basic Blue 124, tetraaminopyrimidine sulfate, and various Disperse or HC series dyes; Tables S10–S12) showed that Phase I remained robust, achieving 91.7% accuracy and correctly identifying the oxidative state in 11 of 12 dyes. By contrast, Phase II performance was lower (9.0% accuracy, 54.5% subset recall), largely due to substitutions of unrepresented colorants with structurally or spectrally analogous compounds. For example, HC Blue No. 15 was misclassified as HC Blue 2 (AX), while HC Red No. 3 (BX) and Basic Violet 2 (IX) were often predicted as GX or FX, reflecting shared chromophore structures and Raman features. These systematic substitutions suggest that DyeSPY attempts to anchor unfamiliar spectra to the closest available analogues within its spectral memory, a behavior consistent with its supervised learning strategy.

These findings underscore both the current boundaries and the promise of DyeSPY. Even without direct spectral matches, the model can infer related chemical structures and return partially correct or chemically relevant predictions. Importantly, these results offer a roadmap for expanding the database: future additions should prioritize frequently substituted or spectrally ambiguous colorants identified during external testing as well the addition of more colorants. Through this adaptive refinement, DyeSPY can evolve into a more comprehensive forensic resource while maintaining the cautious, evidence-driven integrity essential to its application.

Altogether, the cross-study validation demonstrates that DyeSPY’s performance is robust under moderate interstudy variability, particularly in Phase I, and to a lesser extent Phase II, when dye degradation or significant signal distortion is more minimal. However, the system becomes less reliable when the input data diverge too greatly from the training distribution, suggesting that the variability in AuNR synthesis contributes to occasional misclassifications. These results highlight the value of incorporating diverse training spectra, including degraded and variably prepared samples, into future model iterations, ensuring broader applicability for real-world forensic scenarios. Nevertheless, DyeSPY remains a strong first-generation forensic tool capable of triaging unknown samples, identifying likely dye pathways, and excluding incorrect color matches with high confidence.

### 3.8 Limitations

While DyeSPY demonstrates strong accuracy and transferability, several limitations remain. The binary classification of oxidative versus nonoxidative dyes may oversimplify chemical diversity, especially in mislabeled or degraded products that blur these categories. Likewise, the modular architecture depends on Phase I routing; although misclassifications were rare and usually led to inconclusive outputs, they still constrain end-to-end flexibility. Coverage is further limited by the current reference library of 60 dyes, which is a small fraction of the thousands available commercially. Although the system often mapped unfamiliar dyes to close structural analogs or defaulted to “No Color Match”, broader library expansion will be necessary for comprehensive forensic utility.

Additional constraints include potential bias in the oxidative classifier, which was trained on a finite set of authentic formulations, and subjectivity in Phase III perceptual color assignments, which may be affected by lighting, hair porosity, or pigmentation. Finally, the pipeline assumes consistent preprocessing, nanoparticle enhancement, and instrument stability; although cross-study validation confirmed resilience, extreme variability could erode the performance.

Overall, these limitations represent areas for refinement rather than fundamental barriers. The modular design of DyeSPY allows for independent upgrading of each component (i.e., database expansion, routing logic, classifiers, and color metrics), ensuring adaptability as new data and forensic needs emerge.

## 4 Conclusions

The findings of this work demonstrate that SERS, when coupled with machine learning, can transform the forensic analysis of hair dyes from a largely descriptive practice into a chemically precise and interpretable framework. By resolving dye formulations at the colorant level and linking them to perceptual outcomes, DyeSPY provides investigators with information that extends beyond simple color observation, offering insights into identity, chronology, and potential toxicological risks. Importantly, this approach challenges conventional reliance on bulk or morphological methods by emphasizing molecular fidelity and reproducibility, attributes that are critical for courtroom admissibility. Looking forward, the broader significance of this platform lies in its ability to adapt to the variability inherent in real forensic samples, thereby setting the foundation for a standardized, scientifically rigorous tool in forensic hair analysis.

## Supplementary Material



## Notes

Information on colorants used in this study, as well as details of machine learning models with corresponding hyperparameter grids and Python implementation libraries; reaction chemistry; CSNNC-mediated prediction; hair dye products; mean SER spectra; and experimental details (PDF)
