# Supplementary material for: DyeSPY: Establishing the First Forensic SERS Reference for Hair Dye Colorant Evidence
Source: Anal Chem. 2025 Nov 28;97(50):27862–78. doi: 10.1021/acs.analchem.5c05023 (PMC12750408; doi:10.1021/acs.analchem.5c05023)
Supplement: Supplementary file 1 [file ac5c05023_si_001.pdf]

DyeSPY: Establishing the First Forensic SERS Reference for Hair Dye Colorant Evidence

Aidan P. Holman[1,2], Avery Maalouf[1], and Dmitry Kurouski[1,2]*

1. Department of Biochemistry and Biophysics, Texas A&M University, College Station, TX, 77843 United States
2. Interdisciplinary Faculty of Toxicology, Texas A&M University, College Station, TX, 77843 United States

Corresponding author; email: dkurouski@tamu.edu

Table S1. Information on colorants used in this study.

Substance Name	Purity (%)	CAS No.	Supplier	Oxidative?	Type	ESID
N,N-bis(2-hydroxyethyl)-p-phenylenediamine	≥ 97	54381-16-7	Combi Blocks Inc.	Oxidative	Primary Intermediate	A
p-phenylenediamine	≥ 98	106-50-3	Combi Blocks Inc.	Oxidative	Primary Intermediate	B
p-aminophenol	≥ 98	123-30-8	Combi Blocks Inc.	Oxidative	Primary Intermediate	C
2,5-diaminotoluene	≥ 98	95-70-5	Combi Blocks Inc.	Oxidative	Primary Intermediate	D
1-hydroxyethyl-4,5-diamino pyrazole	99.8	155601-30-2	Spectrum Chemical MFG	Oxidative	Primary Intermediate	E
m-aminophenol	≥ 98	591-27-5	Combi Blocks Inc.	Oxidative	Coupler	AA
o-aminophenol	≥ 98	95-55-6	Combi Blocks Inc.	Oxidative	Coupler	BB
2-amino-5-nitrophenol	≥ 98	121-88-0	Combi Blocks Inc.	Oxidative	Coupler	CC
resorcinol	≥ 98	108-46-3	Combi Blocks Inc.	Oxidative	Coupler	DD
4-chlororesorcinol	≥ 98	95-88-5	Combi Blocks Inc.	Oxidative	Coupler	EE
2-methylresorcinol	≥ 98	608-25-3	Cen-Med Enterprises Inc.	Oxidative	Coupler	FF
4-amino-m-cresol	≥ 98	2835-99-6	Combi Blocks Inc.	Oxidative	Coupler	GG
2-amino-3-hydroxypyridine	≥ 98	16867-03-1	Combi Blocks Inc.	Oxidative	Coupler	HH
2-amino-4-methylphenol	≥ 98	95-84-1	Combi Blocks Inc.	Oxidative	Coupler	II
2-methyl-5-hydroxethylaminophenol	≥ 97	55302-96-0	Combi Blocks Inc.	Oxidative	Coupler	JJ
5-amino-6-chloro-o-cresol	≥ 97	84540-50-1	Combi Blocks Inc.	Oxidative	Coupler	KK
3-nitro-p-hydroxyethylaminophenol	≥ 98	65235-31-6	Combi Blocks Inc.	Oxidative	Coupler	LL
2,6-dihydroxyethylaminotoluene	≥ 95	149330-25-6	Combi Blocks Inc.	Oxidative	Coupler	MM
1-naphthol	≥ 98	90-15-3	Combi Blocks Inc.	Oxidative	Coupler	NN
4-amino-2-hydroxytoluene	≥ 98	2835-95-2	Combi Blocks Inc.	Oxidative	Coupler	OO
2,4-diaminophenoxyethanol	≥ 97	66422-95-5	Combi Blocks Inc.	Oxidative	Coupler	PP
2-amino-6-chloro-4-nitrophenol	≥ 98	6358-09-4	Combi Blocks Inc.	Oxidative	Coupler	QQ
2,6-diaminopyridine	≥ 98	141-86-6	Combi Blocks Inc.	Oxidative	Coupler	RR
2,4-diaminopyridine	≥ 98	461-88-1	Combi Blocks Inc.	Oxidative	Coupler	SS
hydroxyethyl-3,4-methylenedioxyaniline	≥ 95	81329-90-0	Combi Blocks Inc.	Oxidative	Coupler	TT
2-amino-4-hydroxyethylaminoanisole	≥ 95	83763-48-8	Combi Blocks Inc.	Oxidative	Coupler	UU
pyrogallol	≥ 98	87-66-1	Combi Blocks Inc.	Oxidative	Coupler	VV
HC Blue 2	≥ 97	33229-34-4	Combi Blocks Inc.	Non-oxidative	Direct Dye	AX
HC Red 3	≥ 97	2871-01-4	Combi Blocks Inc.	Non-oxidative	Direct Dye	BX
HC Red 13	≥ 95	94158-13-1	Combi Blocks Inc.	Non-oxidative	Direct Dye	CX

HC Yellow 4	≥ 98	59820-43-8	Combi Blocks Inc.	Non-oxidative	Direct Dye	DX
HC Yellow 13	≥ 98	10442-83-8	Combi Blocks Inc.	Non-oxidative	Direct Dye	EX
Basic Blue 99	≥ 95	68123-13-7	Combi Blocks Inc.	Non-oxidative	Direct Dye	FX
Basic Brown 16	≥ 95	26381-41-9	Combi Blocks Inc.	Non-oxidative	Direct Dye	GX
Basic Red 51	≥ 95	12270-25-6	United States Biological	Non-oxidative	Direct Dye	HX
Basic Violet 2	≥ 98	3248-91-7	Cen-Med Enterprises Inc.	Non-oxidative	Direct Dye	IX
Acid Red 52	≥ 97	3520-42-1	Combi Blocks Inc.	Non-oxidative	Direct Dye	JX
Acid Red 92	≥ 92.5	18472-87-2	Cen-Med Enterprises Inc.	Non-oxidative	Direct Dye	KX
Basic Violet 3	≥ 90	548-62-9	Sigma-Aldrich	Non-oxidative	Direct Dye	LX
Basic Blue 9	≥ 70	61-73-4	Sigma-Aldrich	Non-oxidative	Direct Dye	MX
Basic Red 14	≥ 90	12217-48-0	Lab Pro Inc.	Non-oxidative	Direct Dye	NX
Basic Violet 4	≥ 98	2390-59-2	Combi Blocks Inc.	Non-oxidative	Direct Dye	OX
Acid Blue 1	≥ 97	116-95-0	Cen-med Enterprises Inc.	Non-oxidative	Direct Dye	PX
Titanium Dioxide	≥ 95	13463-67-7	Combi Blocks Inc.	Non-oxidative	Pigment	IP

Table S2. Information on hair dyes and their colorant ingredients used in this study.

**Product Name**	**Brand**	**Permanence (as Marketed)**	**Colorant (Mixtures)**	**ESID**
Ion Permanent Brights Crème Hair Color Tanzanite	Ion	Permanent	A; KK	D1
Ion Permanent Brights Crème Hair Color 4G Medium Golden Brown	Ion	Permanent	D; AA; DD; FF; PP	D2
Ion Permanent Crème Hair Color 4RV Medium Burgundy Brown	Ion	Permanent	C; D; E; DD; HH; PP	D3
Ion Permanent Crème Hair Color Radiant Orchid	Ion	Permanent	D; E; RR	D4
Ion Permanent Crème Hair Color Garnet	Ion	Permanent	E; TT	D5
Ion Permanent Crème Hair Color Sapphire	Ion	Permanent	A; KK; NN	D6
Ion Permanent Crème Hair Color 1V Jet Black	Ion	Permanent	D; E; AA; FF; PP	D7
Ion Permanent Gloss Hair Color 1B Blue Black	Ion	Permanent	D; PP	D8
Ion Permanent Liquid Hair Color Light Burgundy Brown	Ion	Permanent	C; D; E; DD; HH; PP	D9
Ion Semi-Permanent Crème Hair Color Medium Warm Brown	Ion	Semi-permanent	LL; AX; DX; FX; GX	D10
Ion Semi-Permanent Crème Hair Color Magenta	Ion	Semi-permanent	LL; CX; KX; JX	D11
Ion Semi-Permanent Crème Hair Color Burgundy Brown	Ion	Semi-permanent	LL; AX; BX; GX	D12
Ion Semi-Permanent Crème Hair Color Red	Ion	Semi-permanent	HX	D13
Ion Semi-Permanent Crème Hair Color Blackest Black	Ion	Semi-permanent	AX; DX; FX; GX	D14
Wella Permanent Liquid Hair Color 4NW Medium Neutral Warm Brown	Wella	Permanent	D; AA; DD; FF; NN; OO; UU	D15
Wella Permanent Liquid Hair Color 4RG/347 Dark Auburn	Wella	Permanent	D; DD; GG; NN; OO; QQ	D16
Wella Permanent Gel Hair Color 1N/051 Black	Wella	Permanent	D; AA; OO; PP; TT	D17
Wella Demi-Permanent Hair Color 4N-4/0 Medium Natural Brown	Wella	Demi-permanent	D; AA; FF; TT	D18
Wella Demi-Permanent Hair Color 6W-6/7 Dark Sand	Wella	Demi-permanent	D; FF; OO; QQ; TT	D19
Wella Demi-Permanent Hair Color 1N-2/0 Black	Wella	Demi-permanent	D; DD; UU	D20
Clairol Permanent Liquid Hair Color Light Red Brown	Clairol	Permanent	A; B; C; DD; FF; NN; OO	D21

Clairol Permanent Liquid Hair Color Ultra Cool Black	Clairol	Permanent	A; AA; BB; DD; NN	D22
Clairol Permanent Liquid Hair Color Light Neutral Brown	Clairol	Permanent	A; B; C; AA; DD; FF; NN	D23
L’Oreal Permanent Hair Color Majestic Violet (Dark Violet)	L’Oreal	Permanent	B; AA; DD; OO; PP; JJ	D24
L’Oreal Permanent Hair Color Fresh Ink (Blue Black)	L’Oreal	Permanent	A; D; AA; DD; OO; PP	D25
Schwarzkopf Simply Color Permanent Hair Color 4.68 Chocolate Cherry	Schwarzkopf	Permanent	A; DD; HH	D26
Schwarzkopf Simply Color Permanent Hair Color 4.65 Chestnut Brown	Schwarzkopf	Permanent	A; DD; FF; HH	D27
Schwarzkopf Simply Color Permanent Hair Color 3.0 Darkest Brown	Schwarzkopf	Permanent	A; E; DD; FF; HH	D28
Wella Permanent Liquid Hair Color 3N/311 Dark Brown	Wella	Permanent	D; AA; DD; OO; PP	D29
Wella Permanent Liquid Hair Color 7WV Nutmeg	Wella	Permanent	D; DD; FF; GG; OO; QQ	D30
Wella Permanent Liquid Hair Color 4R/356 Cinnamon Brown	Wella	Permanent	D; AA; DD; FF; OO; QQ	D31
Wella Permanent Liquid Hair Color 9N/911 Very Light Blonde	Wella	Permanent	D, AA, DD, FF, OO	D32
Wella Demi-Permanent Hair Color 10A Lightest Ash Blonde	Wella	Demi-permanent	D; AA; DD; FF; PP	D33
Chi Ionic Shades Hair Color 71 Dark Iridescent Blonde	Chi	Poly-permanent*	C; D; DD; OO; PP; IP	D34
Chi Ionic Shades Hair Color 8RB Medium Red Blonde	Chi	Poly-permanent*	C; D; DD; HH; IP	D35
Chi Ionic Shades Hair Color 4RR Red Plum	Chi	Poly-permanent*	D; E; AA; OO; IP	D36
Chi Ionic Shades Hair Color Red	Chi	Poly-permanent*	E; AA; HH; IP	D37
Schwarzkopf Color Ultimate Permanent Hair Color, 5.29 Vintage Red	Schwarzkopf	Permanent	D; E; AA; OO	D38
Tocco Magico Permanent Hair Color 4R Copper Chestnut	Tocco Magico	Permanent	B; C; EE; FF; HH; OO; UU; DX	D39
Tocco Magico Permanent Hair Color 6.7 Plum	Tocco Magico	Permanent	A; D; EE	D40
Four Reasons Demi-Permanent Hair Color Cherry Tree	Four Reasons	Demi-permanent	B; C; AA; NN; OO	D41
Four Reasons Demi-Permanent Hair Color Black Velvet	Four Reasons	Demi-permanent	A; B; AA; DD; PP; UU	D42
Four Reasons Demi-Permanent Hair Color Coffee Bean	Four Reasons	Demi-permanent	A; B; C; AA; EE; UU	D43
Four Reasons Demi-Permanent Hair Color Caffe Macchiato	Four Reasons	Demi-permanent	B; C; EE; FF; OO; PP; UU	D44
ColorDesign Permanent Hair Color 6NNG Dark Medium Natural Blonde	ColorDesign	Permanent	B; C; D; DD	D45
ColorDesign Permanent Hair Color 5 Light Brown	ColorDesign	Permanent	B; C; AA; DD	D46
ColorEazy Permanent Cream Hair Color 10 Lightest Blonde	ColorEazy	Permanent	B; C; DD; FF	D47
ColorEazy Permanent Cream Hair Color 3RV Medium Auburn	ColorEazy	Permanent	B; C; NN; OO	D48
L'Oreal Technique HiColor Magenta HiLights Permanent Hair Color	L’Oreal	Permanent	HX	D49
Ion Semi-Permanent Crème Hair Color Hottie Pink	Ion	Semi-permanent	IX	D50
Ion Semi-Permanent Crème Hair Color Lemon	Ion	Semi-permanent	DX	D51
Arctic Fox Semi-Permanent Hair Color Wrath	Arctic Fox	Semi-permanent	HX	D52
Arctic Fox Semi-Permanent Hair Color Electric Paradise	Arctic Fox	Semi-permanent	NX	D53
Manic Panic Blue Steel Semi-Permanent Crème Hair Color	Manic Panic	Semi-permanent	FX; MX; OX	D54
Splat Midnight Indigo Moisturizing Semi-Permanent Hair Color	Splat	Semi-permanent	JX; LX; MX; PX	D55
L'Oreal Paris Colo Rista Semi Permanent Color Maroon	L’Oreal	Semi-permanent	AX; HX	D56
Lime Crime Unicorn Semi-Permanent Hair Color Bubblegum Rose	Lime Crime	Semi-permanent	NX	D57
Bleach London Rose Super Cool Semi-Permanent Hair Color	Bleach London	Semi-permanent	KX	D58
Surya Brasil Henna Cream Hair Color Burgundy	Surya Brasil	Semi-permanent	AX; BX; DX	D59

Lime Crime Unicorn Semi-Permanent Hair Color Sour Candy	Lime Crime	Semi-permanent	IX; NX	D60

**Poly-permanent refers to hair dye products that advertise their dye as pre-prepared to color hair without the need for developer, however oxidation is still required in this case and for all intents and purposes, they will be considered oxidative dyes.*

Table S3. Machine learning models with corresponding hyperparameter grids and Python implementation libraries.

Model	Parameter grids	Python libraries
LRDA	Convergence (C): 1, 10, 100<br>Maximum iterations (max_iter): 100, 200, 500<br>Regularization (penalty): L2	scikit-learn
PLSDA	Latent variables (LVs): 2, 3, 4… 20	scikit-learn
RFDA	Maximum tree depth (max_depth): 1, 3, 5<br>Minimum sample splitting (min_samples_split): 2, 5, 10<br>Number of trees (n_estimators): 50, 100, 200, 500	scikit-learn
XGBDA	Learning rate (eta): 0.01, 0.05, 0.001<br>max_depth: 1, 3, 5<br>n_estimators: 50, 100, 200, 500	xgboost, scikit-learn
ANNDA	Layers: [1024, 512], [512, 256]<br>Node dropout (dropout): 0, 0.2, 0.3, 0.5<br>Optimizer: SGD, AdamW, RMSprop<br>Eta: 0.01, 0.001, 0.005, 0.0005<br>Batch sizes: 16, 32, 64<br>L2 weight decay: 0, 0.001<br>L1 weight decay: 0, 0.000001	PyTorch, torch.optim, NumPy, scikit-learn
CSNNC	N/A	NumPy, scikit-learn

N/A: Not applicable

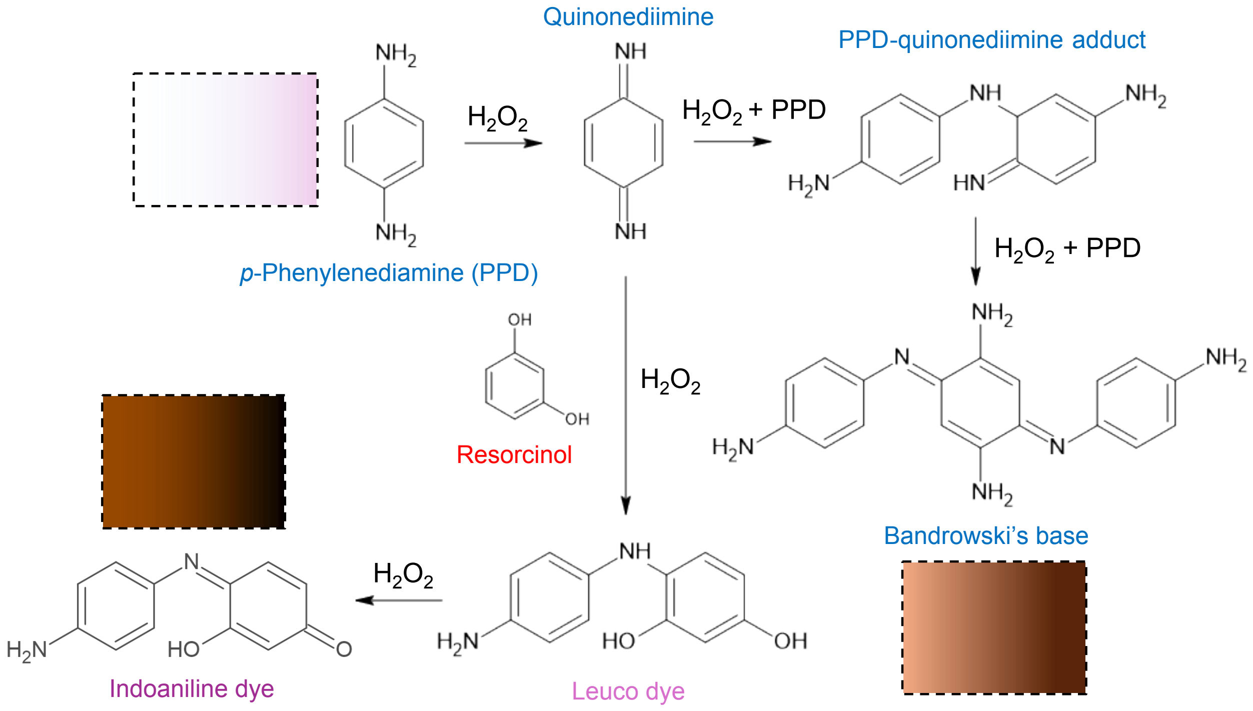

Scheme S1. Reaction chemistry for primaries and couplers. Initially, *p*-phenylenediamine is colorless to light purple. After oxidation it becomes brown to black in color. Couplers, such as resorcinol, can enhance the tone of the color when added, as seen here, or completely change the color.

Table S4. Performance summary of nonoxidative dye mixture classification using synthetic training data generated via Linear Additive Spectral Mixing (LASM) for commercial dye solutions.

ESID(s)	Number of Spectra	True Mixture	(Majority) Predicted Mixture	Proportion of Samples Predicted That, %	Subset recall, %
sD10	5	(LL)+AX+DX+FX+GX	AX+GX	100	100
sD11	5	(LL)+CX+KX+JX	JX	100	100
sD12	5	(LL)+AX+BX+GX	GX	100	100
sD13; sD49; sD52	15	HX	HX	100	100
sD14	5	AX+DX+FX+GX	AX+DX+FX+GX	100	100
sD50	5	IX	IX	100	100
sD52	5	DX	--	100	0
sD53; sD57	10	NX	IX+NX	100	100
sD54	5	FX+MX+OX	FX	60	100
sD55	5	JX+LX+MX+PX	JX+LX+MX+PX	100	100
sD56	5	AX+HX	CX	60	0
sD58	5	KX	--	100	0
sD59	5	AX+BX+DX	GX	100	0
sD60	5	IX+NX	IX+NX	100	100

Hyperparameters: same as in Table 6.

Table S5. CSNNC-mediated prediction of primary intermediates in SER spectra of dyed hair based on primary-labeled primary and dual primary-coupler mixtures (e.g., A+BB = A). Thus, dyes are grouped based on their true mixture ID and not their true primaries. Additionally, the previous prediction is removed from the next possible pool of predictions. Probability of correct primary in 1st prediction = 67.4%; probability of at least one correct primary prediction in first two predictions = 90.7%.

ESID(s)	Number of Spectra	True Primaries	1st Predicted Primary	1st Subset recall, %	2nd Predicted Primary	Final Subset recall, %
hD42	15	A, B	C	0	B	100
hD22	15	A	B	0	A	100
hD23	15	A, B, C	C	100	A	100
hD43	15	A, B, C	B	100	C	100
hD21	15	A, B, C	B	100	C	100
hD25	15	A, D	D	100	C	100
hD27	15	A	B	0	D	0
hD40	15	A, D	B	0	A	100
hD26	15	A, D	B	0	D	100
hD28	15	A, E	C	0	E	100
hD1	15	A	A	100	E	100
hD6	15	A	A	100	E	100
hD24	15	B	B	100	C	100
hD46	15	B, C	B	100	C	100
hD41	15	B, C	A	0	B	100
hD45	15	B, C, D	C	100	B	100
hD47	15	B, C	C	100	E	100
hD39	15	B, C	C	100	B	100
hD44	15	B, C	B	100	C	100
hD48	15	B, C	C	100	B	100

hD35	15	C, D	D	100	C	100
hD34	15	C, D	D	100	C	100
hD3, hD9	30	C, D, E	C	100	D	100
hD15	15	D	D	100	C	100
hD32	15	D	D	100	A	100
hD31	15	D	D	100	A	100
hD33, hD2	30	D	D	100	A	100
hD29	15	D	D	100	E	100
hD18	15	D	D	100	B	100
hD17	15	D	D	100	C	100
hD30	15	D	C	0	A	0
hD16	15	D	D	100	A	100
hD20	15	D	C	0	B	0
hD7	15	D, E	C	0	D	100
hD36, hD38	30	D, E	C	0	D	100
hD4	15	D, E	D	100	E	100
hD19	15	D	C	0	E	0
hD8	15	D	D	100	E	100
hD37	15	E	A	0	E	100
hD5	15	E	E	100	C	100

Table S6. CSNNC-mediated prediction of primary intermediates in SER spectra of commercial hair dyes based on primary-labeled primary and dual primary-coupler mixtures (e.g., A+BB = A). Probability of correct primary in 1st prediction = 81.4; probability of at least one correct primary prediction in first two predictions = 83.7%.

ESID(s)	Number of Spectra	True Primaries	1st Predicted Primary	1st Subset recall, %	2nd Predicted Primary	Final Subset recall, %
hD42	15	A, B	B	100	C	100
hD22	15	A	B	0	D	0
hD23	15	A, B, C	B	100	C	100
hD43	15	A, B, C	B	100	D	100
hD21	15	A, B, C	B	100	C	100
hD25	15	A, D	D	100	C	100
hD27	15	A	D	0	B	0
hD40	15	A, D	B	0	C	0
hD26	15	A, D	D	100	B	100
hD28	15	A, E	B	0	D	0
hD1	15	A	A	100	E	100
hD6	15	A	B	0	E	0
hD24	15	B	B	100	C	100
hD46	15	B, C	B	100	D	100
hD41	15	B, C	C	100	B	100
hD45	15	B, C, D	B	100	C	100
hD47	15	B, C	B	100	A	100
hD39	15	B, C	B	100	C	100
hD44	15	B, C	B	100	C	100
hD48	15	B, C	C	100	B	100
hD35	15	C, D	D	100	C	100
hD34	15	C, D	C	100	B	100
hD3, hD9	30	C, D, E	C	100	C	100
hD15	15	D	D	100	C	100
hD32	15	D	D	100	B	100
hD31	15	D	D	100	B	100
hD33, hD2	30	D	D	100	A	100
hD29	15	D	D	100	B	100
hD18	15	D	D	100	A	100
hD17	15	D	D	100	C	100
hD30	15	D	A	0	C	0
hD16	15	D	D	100	C	100
hD20	15	D	C	0	E	0

hD7	15	D, E	D	100	C	100
hD36, hD38	30	D, E	D	100	C	100
hD4	15	D, E	B	0	D	100
hD19	15	D	D	100	B	100
hD8	15	D	D	100	C	100
hD37	15	E	E	100	C	100
hD5	15	E	E	100	C	100

Table S7. Performance summary of oxidative dye mixture classification using 20-80 train-test partitioning of commercial dye SER spectra.

ESID(s)	Number of Spectra	True Mixture	(Majority) Predicted Mixture	Proportion of Samples Predicted That, %	Subset recall, %
sD1	4	A; KK	A; KK	100	100
sD2, sD33	8	D; AA; DD; FF; PP	D; AA; DD; FF; PP	100	100
sD3, sD9	8	C; D; E; DD; HH; PP	A; D; AA; DD; OO; PP	62.5	100
sD4	4	D; E; RR	D; E; RR	100	100
sD5	4	E; TT	E; TT	100	100
sD6	4	A; KK; NN	A; KK; NN	100	100
sD7	4	D; E; AA; FF; PP	D; E; AA; FF; PP	100	100
sD8	4	D; PP	D; PP	100	100
sD15	4	D; AA; DD; FF; NN; OO; UU	D; AA; DD; FF; NN; OO; UU	100	100
sD16	4	D; DD; GG; NN; OO; QQ	D; DD; GG; NN; OO; QQ	100	100
sD17	4	D; AA; OO; PP; TT	D; AA; OO; PP; TT	100	100
sD18	4	D; AA; FF; TT	D; AA; FF; TT	100	100
sD19	4	D; FF; OO; QQ; TT	D; FF; OO; QQ; TT	100	100
sD20	4	D; DD; UU	D; DD; UU	100	100
sD21	4	A; B; C; DD; FF; NN; OO	A; B; C; DD; FF; NN; OO	100	100
sD22	4	A; AA; BB; DD; NN	A; AA; BB; DD; NN	100	100
sD23	4	A; B; C; AA; DD; FF; NN	A; B; C; AA; DD; FF; NN	100	100
sD24	4	B; AA; DD; OO; PP; JJ	B; AA; DD; OO; PP; JJ	100	100
sD25	4	A; D; AA; DD; OO; PP	A; D; AA; DD; OO; PP	100	100
sD26	4	A; DD; HH	A; DD; HH	100	100
sD27	4	A; DD; FF; HH	A; DD; FF; HH	100	100
sD28	4	A; E; DD; FF; HH	A; E; DD; FF; HH	100	100
sD29	4	D; AA; DD; OO; PP	D; AA; DD; OO; PP	100	100
sD30	4	D; DD; FF; GG; OO; QQ	D; DD; FF; GG; OO; QQ	100	100
sD31	4	D; AA; DD; FF; OO; QQ	D; AA; DD; FF; OO; QQ	100	100
sD32	4	D, AA, DD, FF, OO	D, AA, DD, FF, OO	100	100
sD34	4	C; D; DD; OO; PP; IP	C; D; DD; OO; PP	100	100
sD35	4	C; D; DD; HH; IP	C; D; DD; HH	100	100
sD36, sD38	8	D; E; AA; OO; IP	D; E; AA; OO	100	100
sD37	4	E; AA; HH; IP	E; AA; HH	100	100
sD39	4	B; C; EE; FF; HH; OO; UU; DX	B; C; EE; FF; HH; OO; UU; DX	100	100
sD40	4	A; D; EE	A; D; EE	100	100
sD41	4	B; C; AA; NN; OO	B; C; AA; NN; OO	100	100
sD42	4	A; B; AA; DD; PP; UU	A; B; AA; DD; PP; UU	100	100
sD43	4	A; B; C; AA; EE; UU	A; B; C; AA; EE; UU	100	100
sD44	4	B; C; EE; FF; OO; PP; UU	B; C; EE; FF; OO; PP; UU	100	100
sD45	4	B; C; D; DD	B; C; D; DD	100	100
sD46	4	B; C; AA; DD	B; C; AA; DD	100	100

sD47	4	B; C; DD; FF	B; C; DD; FF	100	100
sD48	4	B; C; NN; OO	B; C; NN; OO	100	100
sNRs	4	NRs	NRs	100	100

Hyperparameters: Layers = [1024, 512], dropout = 0, optimizer = AdamW, eta = 0.001, batch size = 64, L1 = 0, L2 = 0.000001.

Table S8. Hair dye products and their simplified color classification.

ESID	Printed Color	Simplified Color
D1	Tanzanite	Dark Blue
D2	Medium Golden Brown	Yellow Brown
D3	Medium Burgundy Brown	Red Brown
D4	Radiant Orchid	Purple
D5	Garnet	Dark Red
D6	Sapphire	Blue
D7	Jet Black	Black
D8	Blue Black	Dark Blue
D9	Medium Burgundy Brown	Red Brown
D10	Medium Warm Brown	Brown
D11	Magenta	Pink
D12	Light Burgundy Brown	Red Brown
D13	Red	Red
D14	Blackest Black	Black
D15	Medium Neutral Warm Brown	Brown
D16	Dark Auburn	Red Brown
D17	Black	Black
D18	Medium Natural Brown	Brown
D19	Dark Sand	Brown
D20	Black	Black
D21	Light Red Brown	Red Brown
D22	Ultra Cool Black	Black
D23	Light Neutral Brown	Light Brown
D24	Majestic Violet	Dark Purple
D25	Fresh Ink	Dark Blue
D26	Chocolate Cherry	Red Brown
D27	Chestnut Brown	Brown
D28	Darkest Brown	Dark Brown
D29	Dark Brown	Dark Brown
D30	Nutmeg	Brown
D31	Cinnamon Brown	Brown
D32	Very Light Blonde	Light Blonde
D33	Lightest Ash Blonde	Light Blonde
D34	Dark Iridescent Blonde	Blonde
D35	Medium Red Blonde	Red Blonde
D36	Red Plum	Red Purple
D37	Red	Red
D38	Black	Black
D39	Copper Chestnut	Red Brown
D40	Plum	Purple
D41	Cherry Tree	Red
D42	Black Velvet	Dark Red
D43	Coffee Bean	Dark Brown
D44	Caffe Macchiato	Brown
D45	Dark Medium Natural Blonde	Brown
D46	Light Brown	Light Brown
D47	Lightest Blonde	Light Blonde
D48	Medium Auburn	Brown
D49	Magenta	Pink
D50	Hottie Pink	Pink
D51	Lemon	Yellow
D52	Wrath	Dark Red
D53	Electric Paradise	Pink
D54	Blue Steel	Blue
D55	Midnight Indigo	Dark Blue
D56	Maroon	Dark Red

D57	Bubblegum Rose	Light Pink
D58	Rose	Light Pink
D59	Burgundy	Red Brown
D60	Sour Candy	Pink

Table S9. Information on hair dyes and their colorant ingredients used exclusively in Higgins and Kurouski (2023) and Holman et al. (2024).

ESID	Product Name	Colorant(s) in Our Study	Colorant(s) not Included	Color*	Reference Study
hD001_val	Ion Semi-Permanent Hair Color Sapphire	*None*	HC Blue No. 15, Basic Blue 124, Basic Yellow 87	Blue	Higgins and Kurouski (2023) & Holman and Kurouski (2023)
hD002_val	Ion Semi-Permanent Hair Color Radiant Orchid	Basic Violet 2 (IX)	HC Blue No. 15	Purple	Higgins and Kurouski (2023)
hD003_val	Ion Semi-Permanent Hair Color Garnet	Basic Red 51 (HX)	Basic Yellow 87, HC blue No. 15	Red	Higgins and Kurouski (2023)
hD004_val	Ion Permanent Hair Color Magenta	2,6-dihydroxyethylaminotoluene (MM), p-aminophenol (C)	Tetraaminopyrimidine Sulfate	Pink	Higgins and Kurouski (2023)
hD005_val	Wella Semi-Permanent Hair Color Blue	Basic Violet 2 (IX)	HC Blue No. 15, HC Blue No. 16, HC Blue No. 12	Blue	Higgins and Kurouski (2023)
hD006_val	Wella Semi-Permanent Hair Color Wild Orchid	Basic Violet 2 (IX)	HC Blue No. 16, HC Blue No. 12	Purple	Higgins and Kurouski (2023)
hD007_val	Wella Semi-Permanent Hair Color Raspberry	HC Yellow No. 13 (EX), Basic Violet 2 (IX)	HC Blue No. 15, HC Blue No. 16	Pink	Higgins and Kurouski (2023)
hD008_val	Wella Semi-Permanent Hair Color Red	3-nitro-p-hydroxyethylaminophenol (LL), Basic Violet 2 (IX)	HC Red No. 10, HC Red No. 11	Red	Higgins and Kurouski (2023)
hD009_val	L'Oreal Permanent Hair Color 5RV Chroma Ruby	4-amino-2-hydroxytoluene (OO), 2-methyl-5-hydroxyethylaminophenol (JJ), titanium dioxide (IP), resorcinol (DD), p-aminophenol (C), Diamino toluene (D),	Hydroxyethoxy aminopyrazolopyridine HCl	Red	Higgins and Kurouski (2023)
hD010_val	Clairol Semi-Permanent Hair Color B09W Light Reddish Brown	HC Red No. 3 (BX)	HC Yellow No. 2, HC Orange No. 1, Disperse Black 9, Disperse Violet 1	Auburn	Higgins and Kurouski (2023)
hD011_val	Clairol Semi-Permanent Hair Color B13W Medium Warm Brown	HC Blue No. 2 (AX), HC Red No. 3 (BX)	HC Yellow No. 2, Disperse Blue 377, Disperse Violet 1	Brown	Higgins and Kurouski (2023)
hD012_val	Clairol Semi-Permanent Hair Color B22D Jet Black	HC Blue No. 2 (AX), HC Red No. 3 (BX)	Disperse Blue 377, Disperse Black 9, HC Yellow No. 2, Disperse Violet 1	Black	Higgins and Kurouski (2023)

**Colors were pulled from the relevant reference studies since these hairs were not dyed in this study nor using the same hair.*

Table S10. Cross-study validation of DyeSPY using raw SER spectra of dyed hair groups that were not included in this study but produced from Higgins and Kurouski (2023), utilizing the DyedHairModules pipeline.

ESID	Number of Spectra	Phase I Prediction	Phase I Actual	Phase II Prediction	Phase II Actual	Phase III Prediction	Phase III Actual
hD001_val	50	Nonoxidative (94%)[a]	Nonoxidative	CX (94%)	*None (0%)*	No Color Match (100%)	Blue
hD002_val	50	Nonoxidative (100%)	Nonoxidative	AX (54%)	IX (32%)	No Color Match (76%)	Purple
hD003_val	50	Nonoxidative (100%)	Nonoxidative	AX+FX+HX (100%)	HX	No Color Match (60%)	Red
hD004_val	50	Oxidative (92%)	Oxidative	A+KK+NN (88%)	C+MM (0%)	No Color Match (100%)	Pink
hD005_val	50	Nonoxidative (100%)	Nonoxidative	FX (100%)	IX	No Color Match (100%)	Blue
hD006_val	50	Nonoxidative (100%)	Nonoxidative	FX (100%)	IX	No Color Match (100%)	Purple
hD007_val	50	Nonoxidative (100%)	Nonoxidative	IX+NX (92%)	EX+IX (0%)	Pink (56%)	Pink
hD008_val	50	Nonoxidative (100%)	Nonoxidative	AX+DX+FX+GX (100%)	IX+LL	No Color Match (100%)	Red
hD009_val	50	Nonoxidative (96%)	Oxidative (4%)	*None (72%)*	C+D+DD+OO+JJ+IP (0%)	No Color Match (100%)	Red
hD010_val	50	Nonoxidative (96%)	Nonoxidative	AX+GX (62%)	BX (0%)	No Color Match (100%)	Auburn
hD011_val	50	Nonoxidative (100%)	Nonoxidative	DX+FX+GX (100%)	AX+BX	No Color Match (100%)	Brown
hD012_val	50	Nonoxidative (100%)	Nonoxidative	AX+DX+FX+GX (98%)	AX+BX (0%)	No Color Match (100%)	Black
**Total Samples**	**Total Spectra**	**Phase I Accuracy[b]**	**Phase II Accuracy[b,c,d]**	**Phase II Nonoxidative Subset recall[b,d]**	**Phase II Oxidative Subset recall[b,d]**	**Phase II Subset recall[b,d]**	**Phase III Accuracy[b,c,d]**
12	600	91.7%	9.0%	40%	0%	33.3%	100%

a: Percentages indicate the proportion of spectra predicted in that category; b: Calculated at the sample level; spectral percentages do not affect these values. IC is treated as incorrect; c: Phase II Accuracy = (correct colorants ÷ total predicted colorants), averaged across samples; d: Phase II and III metrics include only samples correctly classified in Phase I; e: Phase III Accuracy counts "No Color Match" as correct for incorrect colorant predictions, but incorrect when true colorants are correctly predicted.

Table S11. Cross-study validation of DyeSPY using raw SER spectra of dyed hair groups that were <u>not included</u> in this study but produced from Holman and Kurouski (2023), utilizing the DyedHairModules pipeline.

ESID	Number of Spectra	Weeks in the Sun	Phase I Prediction	Phase I Actual	Phase II Prediction	Phase II Actual	Phase III Prediction	Phase III Actual
hD001_val	50	0	Nonoxidative (100%)[a]	Nonoxidative	*None (64%)*	*None*	No Color Match (100%)	Blue
	50	1	Nonoxidative (98%)	Nonoxidative	GX (84%)	*None (6%)*	No Color Match (100%)	Blue

	50	2	Nonoxidative (100%)	Nonoxidative	*None (86%)*	*None*	No Color Match (100%)	Blue
	50	3	Nonoxidative (100%)	Nonoxidative	*None (74%)*	*None*	No Color Match (100%)	Blue
	50	4	Nonoxidative (100%)	Nonoxidative	*None (58%)*	*None*	No Color Match (100%)	Blue
	50	5	Oxidative (96%)	Nonoxidative (4%)	A+B+C+AA+DD+FF+NN (96%)	*None (2%)*	No Color Match (100%)	Blue
	50	6	Nonoxidative (80%)	Nonoxidative	IC	*None (32%)*	No Color Match (100%)	Blue
	50	7	Nonoxidative (96%)	Nonoxidative	AX+GX (54%)	*None (2%)*	No Color Match (100%)	Blue
	50	8	Nonoxidative (70%)	Nonoxidative	*None (70%)*	*None*	No Color Match (100%)	Blue
	50	9	Nonoxidative (84%)	Nonoxidative	*None (82%)*	*None*	No Color Match (100%)	Blue
	50	10	Oxidative (96%)	Nonoxidative (4%)	E+TT (80%)	*None*	No Color Match (100%)	Blue
	**Samples**	**hD001_val Spectra**	**hD001_val Phase I Accuracy[b]**		**hD001_val Phase II Accuracy[b,c,d]**	**hD001_val Phase II Subset recall[b,d]**	**hD001_val Phase III Accuracy[b,c,d]**	
	11	550	81.8%		54.5%	*54.5%*	100%	

IC: Inconclusive for predictions with ≤50% spectral support; a: Percentages indicate the proportion of spectra predicted in that category; b: Calculated at the sample level; spectral percentages do not affect these values. IC is treated as incorrect; c: Phase II Accuracy = (correct colorants ÷ total predicted colorants), averaged across samples; d: Phase II and III metrics include only samples correctly classified in Phase I; e: Phase III Accuracy counts "No Color Match" as correct for incorrect colorant predictions, but incorrect when true colorants are correctly predicted.

Table S12. Cross-study validation of DyeSPY on visually degraded SER signal of dyes on hair from Holman and Kurouski (2023).

ESID	Number of Spectra	Weeks in the Sun	Phase I Prediction	Phase I Actual	Phase II Prediction	Phase II Actual	Phase III Prediction	Phase III Actual
D1	50	8	Oxidative (100%) [a]	Oxidative	E+TT (100%)	A+KK	No Color Match (100%)	Dark Blue
	50	9	Oxidative (98%)	Oxidative	E+TT (80%)	A+KK (0%)	No Color Match (100%)	Dark Blue
	50	10	Oxidative (100%)	Oxidative	E+TT (96%)	A+KK (4%)	No Color Match (100%)	Dark Blue
D7	50	8	Oxidative (96%)	Oxidative	E+TT (92%)	D+E+AA+FF+PP (0%)	No Color Match (100%)	Black (20%)
	50	9	Oxidative (98%)	Oxidative	E+TT (98%)	D+E+AA+FF+PP (0%)	No Color Match (100%)	Black (32%)
	50	10	Oxidative (100%)	Oxidative	E+TT (96%)	D+E+AA+FF+PP (0%)	No Color Match (96%)	Black

D14	50	8	Oxidative (100%)	Nonoxidative	E+TT (100%)	AX+DX+FX+GX	No Color Match (100%)	Black (20%)
	50	9	Nonoxidative (94%)	Nonoxidative	AX+DX+FX (62%)	AX+DX+FX+GX (16%)	No Color Match (100%)	Black
	50	10	Nonoxidative (100%)	Nonoxidative	FX (68%)	AX+DX+FX+GX (20%)	Brown (70%)	Black

***a**: The percentages represent the number of spectra predicted that way.*

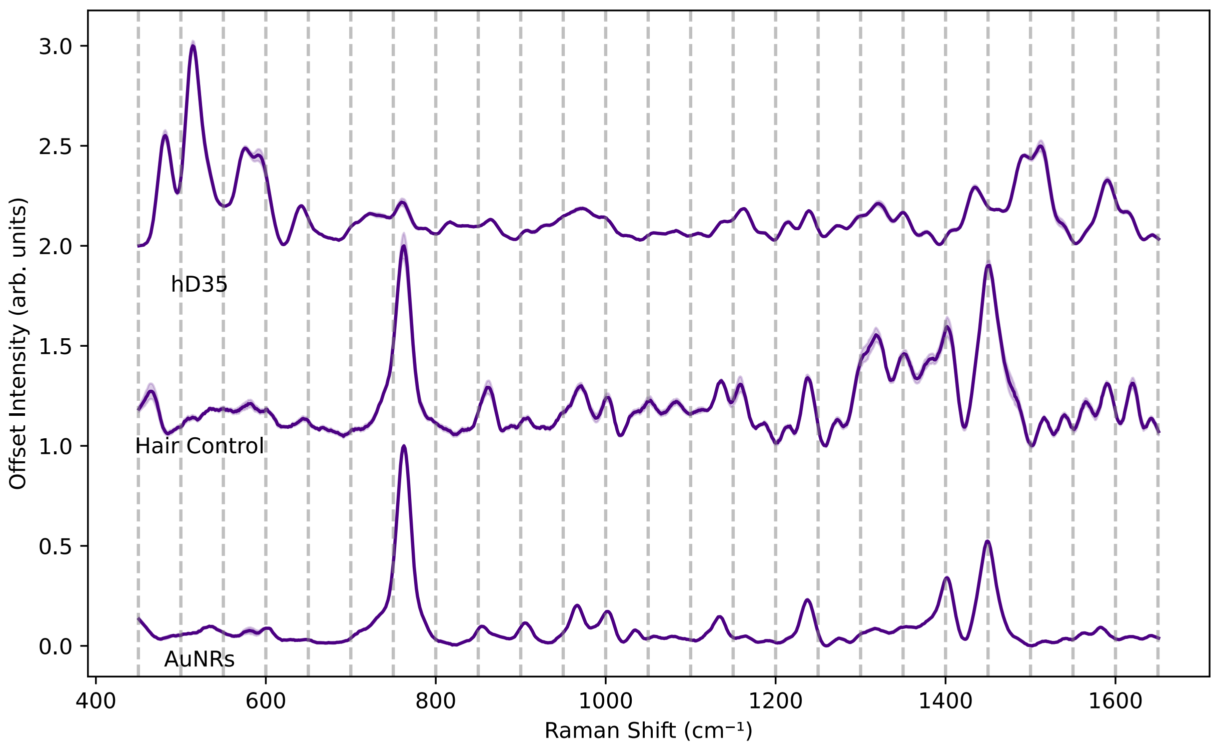

Figure S1. Mean SER spectra and SE of AuNRs on glass coverslip, AuNRs on virgin (undyed) hair (Hair Control), and an example of dyed hair (hD35) to show where AuNR signal aligns.

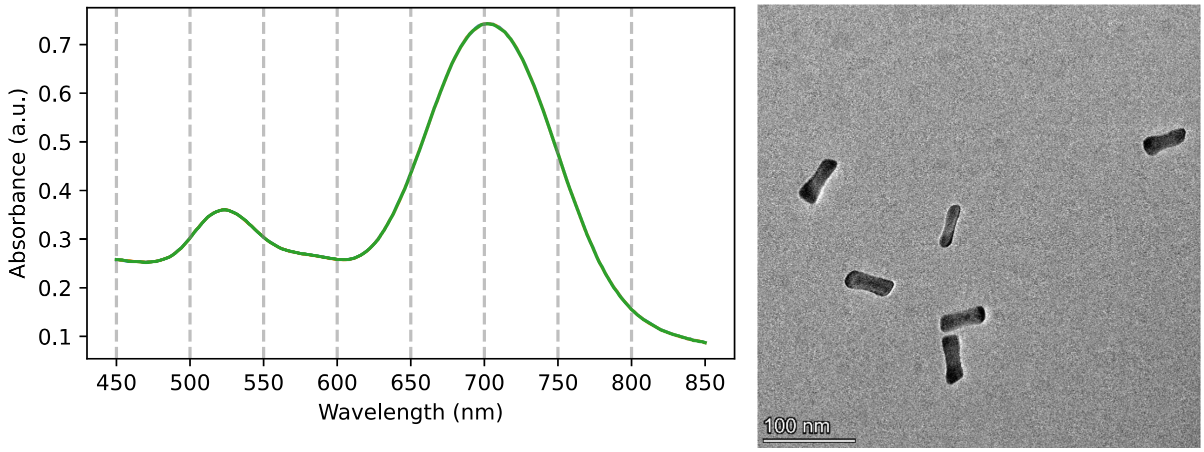

Figure S2. (Left) Mean and SE raw UV-Vis spectrum and (right) transmission electron micrograph of AuNRs. Maxima are located at 522.7 and 702.7 nm in the UV-Vis spectrum.

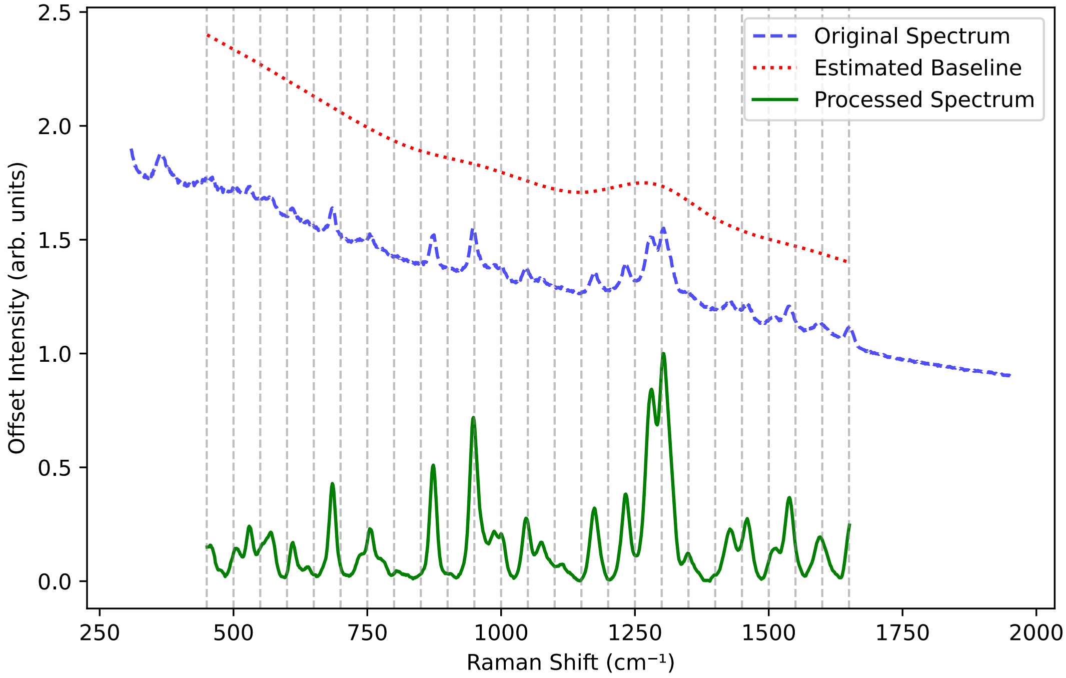

Figure S3. Visualization of preprocessing effects. The gray dashed lines extend the length of the trim: 450 to 1650 cm^{-1}.

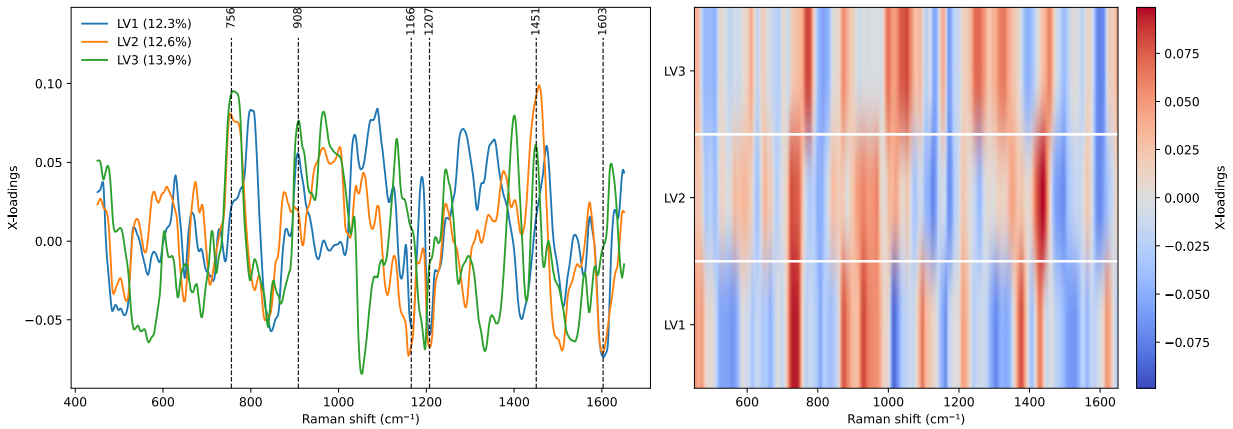

Figure S4. PLSDA loading profiles (left) for the first three latent variables (LV1–LV3), which cumulatively account for 38.8% of the variance in Phase I oxidative/nonoxidative classification, and corresponding loading heatmap (right) illustrating the relative sign and magnitude of spectral contributions across the Raman domain.

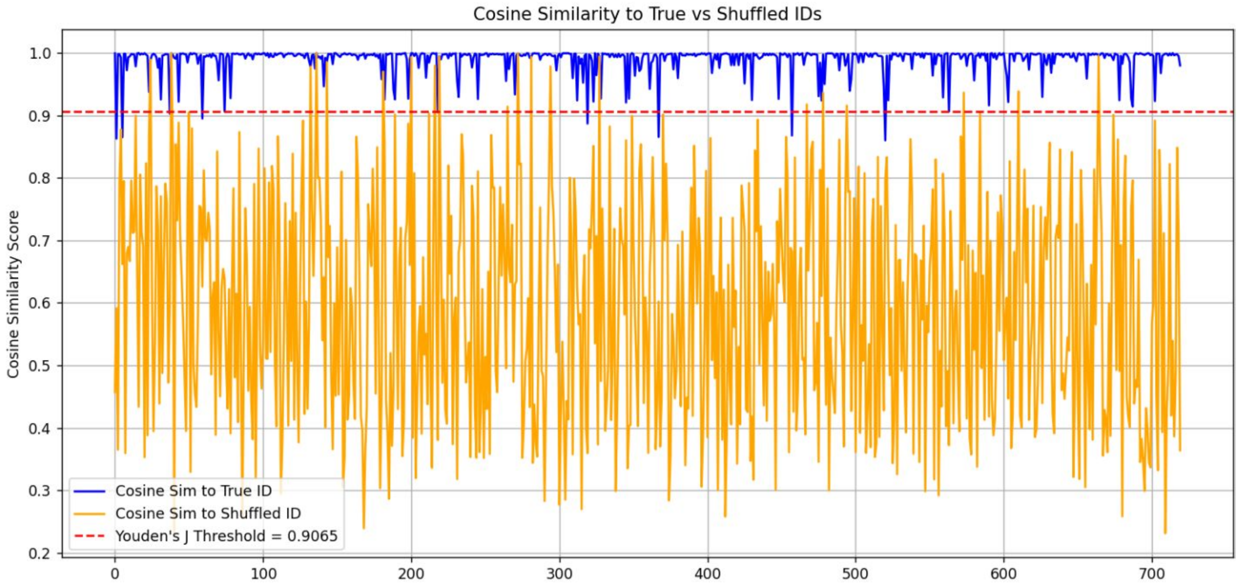

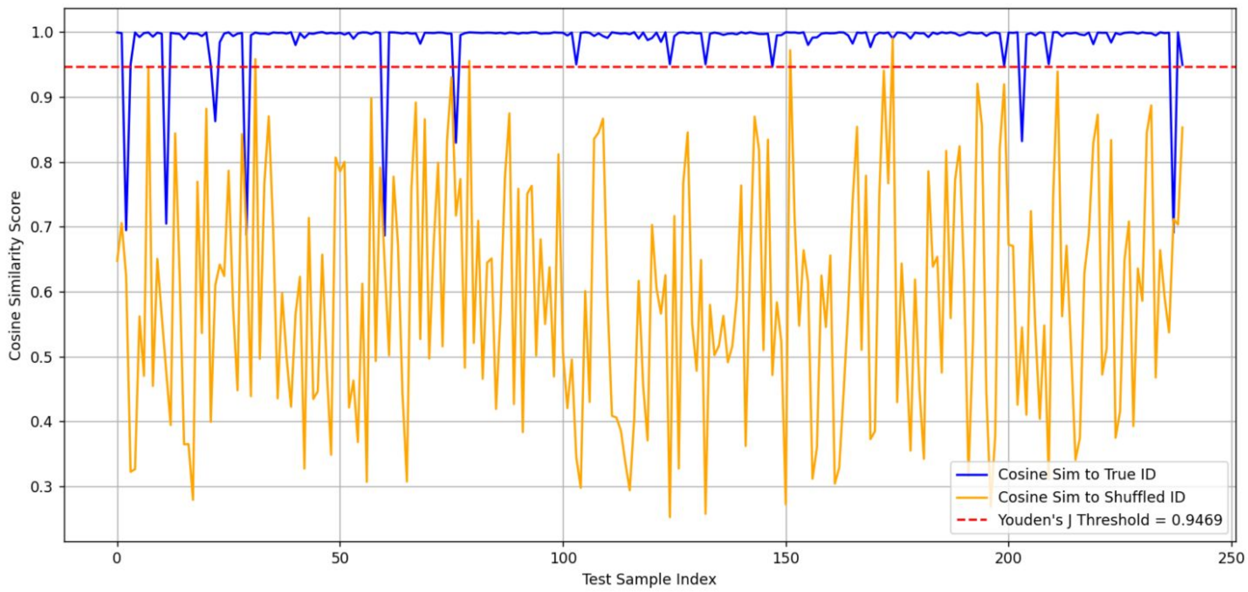

Figure S5. Cosine similarity scores for each test sample in the (top) dyed hair and (bottom) dye solution datasets. Blue lines indicate cosine similarity between each test spectrum and its corresponding true mixture ID (correct class), while orange lines show cosine similarity to randomly shuffled mixture IDs (false matches). The red dashed line represents the Youden's J threshold, which maximizes the difference between true positive and false positive rates, serving as the decision boundary for class match acceptance.
